# The identification and deletion of the polyketide synthase‐nonribosomal peptide synthase gene responsible for the production of the phytotoxic triticone A/B in the wheat fungal pathogen *Pyrenophora tritici‐repentis*

**DOI:** 10.1111/1462-2920.14854

**Published:** 2019-11-21

**Authors:** Catherine Rawlinson, Pao Theen See, Paula Moolhuijzen, Hang Li, Caroline S. Moffat, Yit‐Heng Chooi, Richard P. Oliver

**Affiliations:** ^1^ Centre for Crop and Disease Management Curtin University Perth 6150 Western Australia Australia; ^2^ School of Molecular Sciences The University of Western Australia Perth 6009 Western Australia Australia

## Abstract

The economically important necrotrophic fungal pathogen, *Pyrenophora tritici‐repentis* (Ptr), causes tan spot of wheat, a disease typified by foliar necrosis and chlorosis. The culture filtrate of an Australian Ptr isolate, M4, possesses phytotoxic activity and plant bioassay guided discovery led to the purification of necrosis inducing toxins called triticone A and B. High‐resolution LC–MS/MS analysis of the culture filtrate identified an additional 37 triticone‐like compounds. The biosynthetic gene cluster responsible for triticone production (the *Ttc* cluster) was identified and deletion of *TtcA*, a hybrid polyketide synthase (PKS)‐nonribosomal peptide synthase (NRPS), abolished production of all triticones. The pathogenicity of mutant (*ttcA*) strains was not visibly affected in our assays. We hypothesize that triticones possess general antimicrobial activity important for competition in multi‐microbial environments.

## Introduction

At least 15 000 specialized or secondary metabolites have been identified in ascomycete fungi and many have been shown to have important roles in signalling, defence or as toxins (Losada *et al*., 2009; Spiteller, 2015; Macheleidt *et al*., 2016; Wakefield *et al*., 2017; Künzler, 2018; Chapman and Hall, 2019). The biosynthesis of these specialized metabolites is generally facilitated by any of four core biosynthetic enzymes; polyketide synthase (PKS), non‐ribosomal peptide synthase (NRPS), terpene synthase (TPS) or dimethylallyl tryptophan synthase (DMATS) (Keller *et al*., 2005) or are ribosomally synthesized and post‐translationally modified (Hetrick and van der Donk, 2017). In some cases, two or more synthases are used (Boettger and Hertweck, 2013; Fisch, 2013). For example, the cytochalasins, metabolites that contain both ketone and amide functional groups, are synthesized via a PKS‐NRPS hybrid (Scherlach *et al*., 2010; Qiao *et al*., 2011). The genes encoding synthases are normally clustered contiguously with additional genes encoding further biosynthetic modifications, transport, regulatory and self‐protection functions (Walsh, 2008; Keller, 2015).

*Pyrenophora tritici‐repentis* (Ptr) is a dothideomycete fungal pathogen and the causal agent of tan spot, an economically important and world‐wide foliar disease of wheat. Three host specific toxins, the proteinaceous effectors ToxA and ToxB and the semi‐characterized small molecule effector ToxC have been described for Ptr (Effertz *et al*., 2002; Ciuffetti *et al*., 2010; Lamari and Strelkov, 2010). In addition, two classes of specialized metabolites, anthraquinones and triticones, have been reported as non‐specific phytotoxins (Sugawara *et al*., 1988; Hallock *et al*., 1993; Kachlicki and Wakulinski, 2002; Bouras and Strelkov, 2008). Anthraquinones are characterized by three fused aromatic rings and harbour a variety of substituents. They are produced by many endophytic fungi, have a broad range of biological activities and their biosynthesis has been well studied (Thomas, 2001; Gessler *et al*., 2013). Four anthraquinones, emodin, catenarin, helminthosporin, islandicin and their hydroxylated forms have been detected in culture filtrates, infected leaves and grains of wheat (Kachlicki and Wakulinski, 2002; Wakuliński *et al*., 2003). Catenarin is thought to be the cause of red smudge, a red discolouration of grains predominantly found on infected durum wheat (Bouras and Strelkov, 2008).

The triticones, also known as spirostaphylotrichins, are characterized by a spirocyclic γ‐lactam core structure. Triticones A to F were first purified from Ptr culture filtrates but have since been found, along with an additional 18 other triticone compounds, in 5 other ascomycete fungi, *Staphylotrichum coccosporum*, *Curvularia pallescens*, *Pyrenophora seminiperda*, *Cochliobolus lunatus* and *Bipolaris* spp. (Sugawara *et al*., 1988; Sandmeier and Tamm, 1989a,b, 1990; Hallock *et al*., 1993; Abraham *et al*., 1995a; de Almeida *et al*., 2017; Wang *et al*., 2018). Triticone A and B are known to be phytotoxic, producing yellowish‐brown lesions following leaf puncture assays on a range of hosts including wheat. Triticone C and D were described as weakly active and triticones E and F as inactive (Hallock *et al*., 1993). It was postulated that triticone A alters host photosynthetic processes via inhibition of thiol‐containing enzymes (Kenfield *et al*., 1988). Biosynthesis of triticones in Ptr has not been pursued and the biosynthetic pathway remains unknown. Recently, a biosynthetic gene cluster (*Cpa*) containing a PKS‐NRPS hybrid gene (*CpaA*) from the endophytic fungus *C. pallescens* (DSM62482) was shown to produce a class of structurally similar compounds called curvupallides (Yokoyama *et al*., 2017). Yokoyama (2017) proposed that spirostaphylotrichin C (triticone A) is also derived from *CpaA*.

Our objective was to purify and characterize small molecules which might contribute to virulence in culture filtrates of Ptr. In the current study, we present the plant bioassay‐led purification of triticones A and B and identify a further 37 triticone‐like compounds. The biosynthetic gene cluster was identified and deletion of the core PKS‐NRPS biosynthetic gene prevented production. However, virulence was not obviously affected.

## Results

### *Purification of a small molecule causing necrosis on wheat leaves*

**Figure 1:**
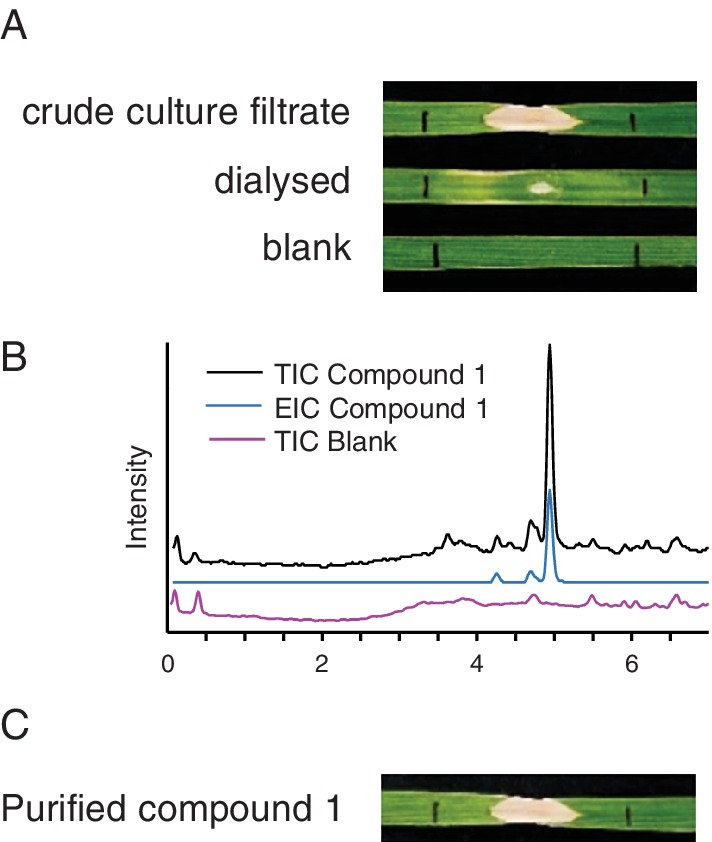
Necrosis inducing small molecule. A. Infiltration of crudefiltrate, dialysed culture filtrate of *Ptr* isolate, M4 and a water dialysed blank into wheat. B. LC‐Q‐MS analysis displaying the TIC and EIC(m/z 278) of purified Compound **1** and the TIC of the preceding solvent blank. C. Infiltration of purified necrosis‐inducing small molecule into wheat. [Color figure can be viewed at http://wileyonlinelibrary.com]

Infiltration of the culture filtrate of Ptr M4, a ToxA and ToxC producing Ptr isolate, into leaves of the ToxA insensitive wheat cultivar Eagle Rock produced a beige ovoid region of necrosis which did not extend to the entire infiltration zone (Fig. 1A). When the culture filtrate was dialysed using a <3.5 kDa cut‐off, necrotic activity was heavily reduced (Fig. 1A). A combination of dispersive solid phase extraction (dSPE) and iterative high‐performance liquid chromatography (HPLC) attached to a diode array detector (DAD) was used to fractionate the culture filtrate and all fractions were analysed via LC‐Q‐MS and infiltrated into wheat (see document Supporting Information, Fig. S1/S2 for details). The fraction exhibiting highest necrotic activity had two major components both with an *m/z* of 278. Final purification led to the isolation of compound **1** as an off white powder with an [M + H]^+^ of 278 (Fig. 1B), which produced the beige ovoid necrotic symptom (Fig. 1C).

### *Identification of phytotoxin triticone A/B (1) and C/D (2) in Ptr*

**Figure 2:**
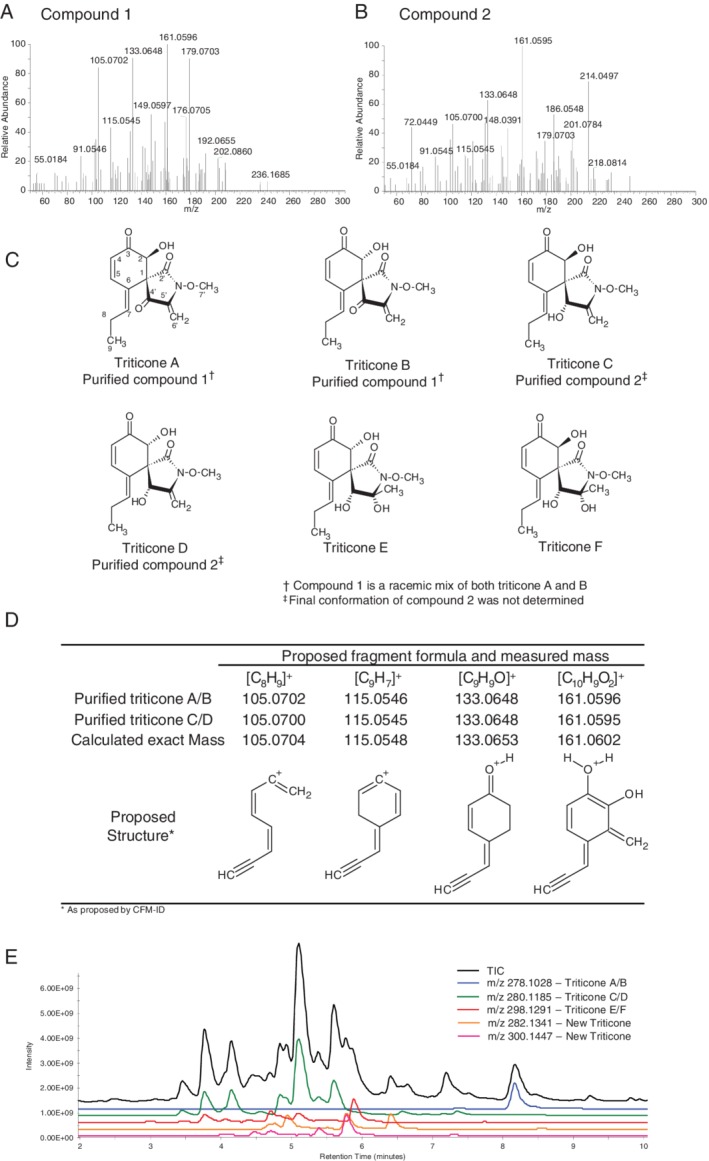
The triticones of *Ptr*. A. accurate mass MS/MS spectra of **1**f. B. accurate mass MS/MS spectra of **2**. C. Characterized triticones previously identified in *Ptr* culture filtrate by Sugawara (1988) and Hallock (1993). D. Common fragment ions in 1 and 2 and their proposed molecular structure. E. Total ion chromatogram and extracted ion chromatograms from M4 crude culture filtrate of all triticone precursor ions within a 5 ppm window. [Color figure can be viewed at http://wileyonlinelibrary.com]

High‐resolution LC–MS/MS analysis of **1** revealed a precursor ion mass of *m/z* 278.1023. The MS/MS spectra of **1** (Fig. 2A) contained fragment ions in common with 37 other features in the crude culture filtrate. To determine whether a similar core structure was present in these features a second, but inactive compound (**2**), containing a similar fragmentation pattern (Fig. 2B) was purified. Compound **2** was purified using the same protocol as **1** (Supporting Information Fig. S3) and had a measured accurate mass of *m/z* 280.1178.

The accurate mass precursor ions for **1** and **2** were consistent with the calculated mass of triticone A or B and triticone C or D, isomer pairs of small molecule phytotoxins previously characterized in Ptr (Fig. 2C) (Hallock *et al*., 1993). The molecular formula of triticone A/B is C_14_H_15_NO_5_, with a calculated [M + H]^+^ mass of *m/z* 278.1028. Triticone C/D has a molecular formula of C_14_H_17_NO_5_ and calculated [M + H]^+^ mass of *m/z* 280.1185. Comparison of calculated versus experimental accurate masses showed a −1.8 ppm error for **1** as triticone A or B and −2.5 ppm error for **2** as triticone C or D. Proton and carbon NMR analyses indicated that **1** was a 100:80 mix of two analytes with the same proton and carbon signals with slightly offset chemical shifts indicative of a mixture of isomers (Supporting Information Figs. S4 and S5). Comparison of the proton and carbon NMR to published data (Hallock *et al*., 1993) confirmed the identification of **1** as a mix of the interconvertible triticone A and B (Supporting Information Table S1) and **2** as either triticone C or D (Hallock *et al*., 1993) (Supporting Information Figs. S6 and S7 and Table S2).

### *Detection of novel triticones in the culture filtrate of Ptr*

Competitive Fragmentation Modelling for Metabolite Identification (CFM‐ID, 2016), a web‐based tool for MS/MS fragment structure prediction, proposed the molecular structures for four fragments of both **1** and **2** (Fig. 2D) of *m/z* 105.0704, 115.0548, 133.0653 and 161.0602, which were used to scout the high‐resolution LC–MS/MS profile of the M4 culture filtrate for triticones E/F and any previously uncharacterized triticone candidates. This identified a total of 38 triticone candidates, including **1** and **2**. Of the 38 components, 23 candidates contained a precursor ion mass within 5 ppm of the 6 known triticones produced by Ptr (Fig. 2E, see Triticone_MSMS.mgf in Supporting Information for MS/MS library of all triticone candidates). These 23 candidates comprised 2 compounds consistent with the molecular formula of triticone A/B (C_14_H_15_NO_5_), 12 compounds consistent with triticone C/D (C_14_H_17_NO_5_), and 9 compounds consistent with triticone E/F (C_14_H_19_NO_6_). An additional 15 candidates were identified with precursor ion masses not previously characterized in Ptr. Nine compounds had an exact mass consistent with a C_14_H_19_NO_5_ formula and average measured mass of *m/z* 282.1335, a −2.2 ppm error on the calculated accurate mass. Six compounds had a predicted molecular formula of C_14_H_21_NO_6_ with an average measured mass of *m/z* 300.1440, a −2.2 ppm error.

### *Ptr culture filtrate LC–MS profiling shows variable triticone production*

High‐resolution LC–MS analysis identified over 1100 features in the filtrate of M4 of which the 38 triticone compounds accounted for 30% of the total intensity of all measured compounds. To investigate the prevalence of triticones in different Ptr isolates, the crude culture filtrates of isolates 239, 134, 11,137, 5213 and V1 from Australia and DW5 and DW7 from the United States (Supporting Information Fig. S8A) were analysed via LC–MS. No additional triticones were found in any isolate on top of the 38 found in M4. The production of triticones in isolates 239, 134, 11,137, DW5 and DW7 was as diverse as M4 and included all 38 triticone candidates. Isolate 5213 produced 24 and V1 produced only 2 triticone candidates, compound **2** along with an unknown with the formula of triticone A/B. The relative raw intensities of individual triticones varied markedly (Supporting Information Table S3). Isolates that produced the greatest diversity of triticones (M4, 239, 134, 11,137, DW5 and DW7) also produced the greatest total amount. Infiltration of the crude culture filtrate of individual isolates into Eagle Rock induced necrosis that increased in size with increasing **1** concentration (Supporting Information Figs. S8B and S8C).

### *Compound 1 (triticone A/B) exhibits low‐level phytotoxicity on host and non‐host plants*

**Figure 3:**
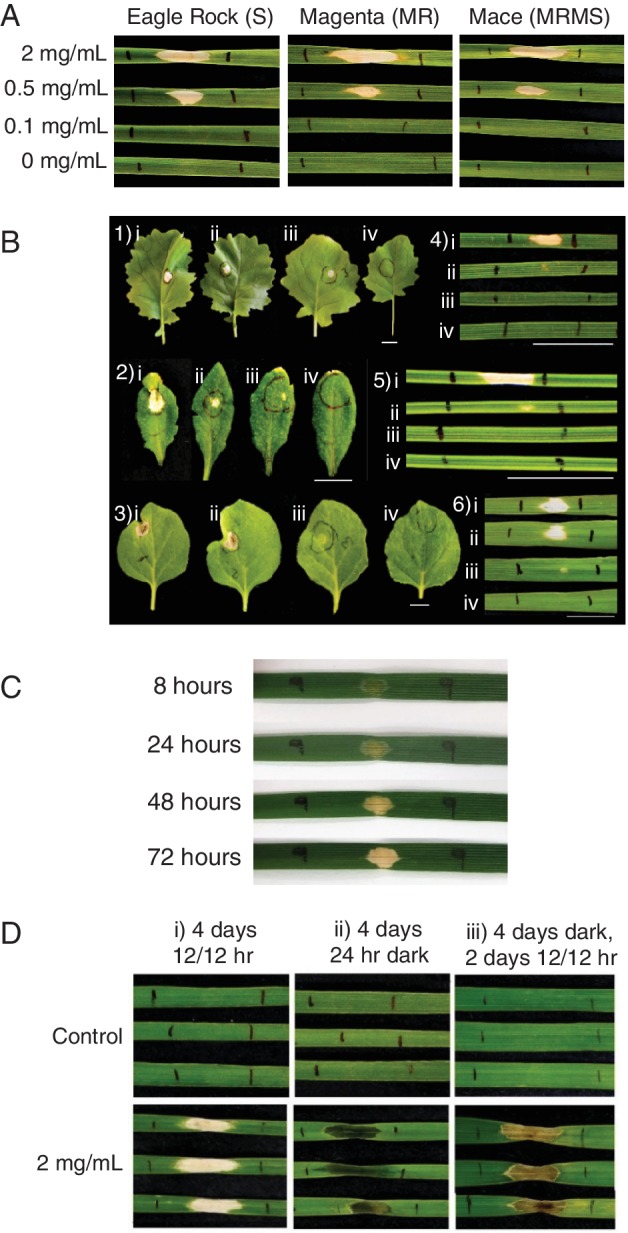
Activity of triticone A/B. A. Infiltration of **1** in to 3 wheat cultivars of differing tan spot susceptibility at 2, 0.5, 0.1 mg ml^−1^ and 0 mg ml^−1^. B. Infiltration in to 1) Canola, 2) Arabidopsis, 3) Nicotiana, 4) Brachypodium, 5) Ryegrass and 6) Barley at i) 2.0 mg ml^−1^, ii) 0.5 mg ml^−1^, iii) 0.1 mg ml^−1^ and iv) 0 mg ml^−1^ (white bar represents 1 cm). C. Development of necrosis symptom produced by **1** at 8, 24, 48 and 72 h post infiltration. D. Infiltration of **1** at 2.0 mg ml^−1^ in to Eagle Rock and placed in i) 4 days of 12/12 h day/night cycle, ii) 4 days of 24 h night and iii) 4 days of 24 h night followed by 2 days of 12/12 h day/night cycle. [Color figure can be viewed at http://wileyonlinelibrary.com]

Compound **1** was infiltrated at 2.0, 0.5 and 0.1 mg ml^−1^ in to three wheat cultivars (Fig. 3A) represented by cultivars rated as susceptible (S), moderately resistant/moderately susceptible (MRMS) and moderately resistant (MR) to tan spot (Eagle Rock, Magenta and Mace respectively) (DPIRD, 2018). Necrotic symptoms were comparable across cultivars, were smaller when infiltrated with 0.5 mg ml^−1^ and no activity was seen at 0.1 mg ml^−1^. Additional plant species were infiltrated to test host specificity including the monocots *Brachypodium distachyon*, ryegrass and barley and the dicots canola, *Arabidopsis thaliana* and tobacco. All showed comparable responses to wheat (Fig. 3B), exhibiting necrotic symptoms at 2 and 0.5 mg ml^−1^ with minimal to no activity at 0.1 mg ml^−1^.

### *Compound 1 (triticone A/B) induced light‐sensitive necrosis on wheat leaves*

Compound **1** was infiltrated at 2 mg ml^−1^ into wheat and incubated under three light settings to investigate the light dependence of necrotic activity. Immediately upon infiltration a translucent zone was visible, which, under 12/12‐h light/dark cycle, developed to the highly localized beige‐coloured necrotic region after 72 h [Fig. 3C and D (i)]. When incubated in complete darkness for 4 days, infiltrated leaves did not progress beyond a translucent zone [Fig. 3D (ii)]. However, when kept in darkness for 4 days and then placed in 12/12 light/dark cycle for 2 days the translucent zone developed to a darker, brown‐coloured necrotic symptom [Fig. 3D (iii)].

### *Identification of the PKS‐NRPS hybrid gene in M4 genome responsible for triticone production*

**Figure 4:**
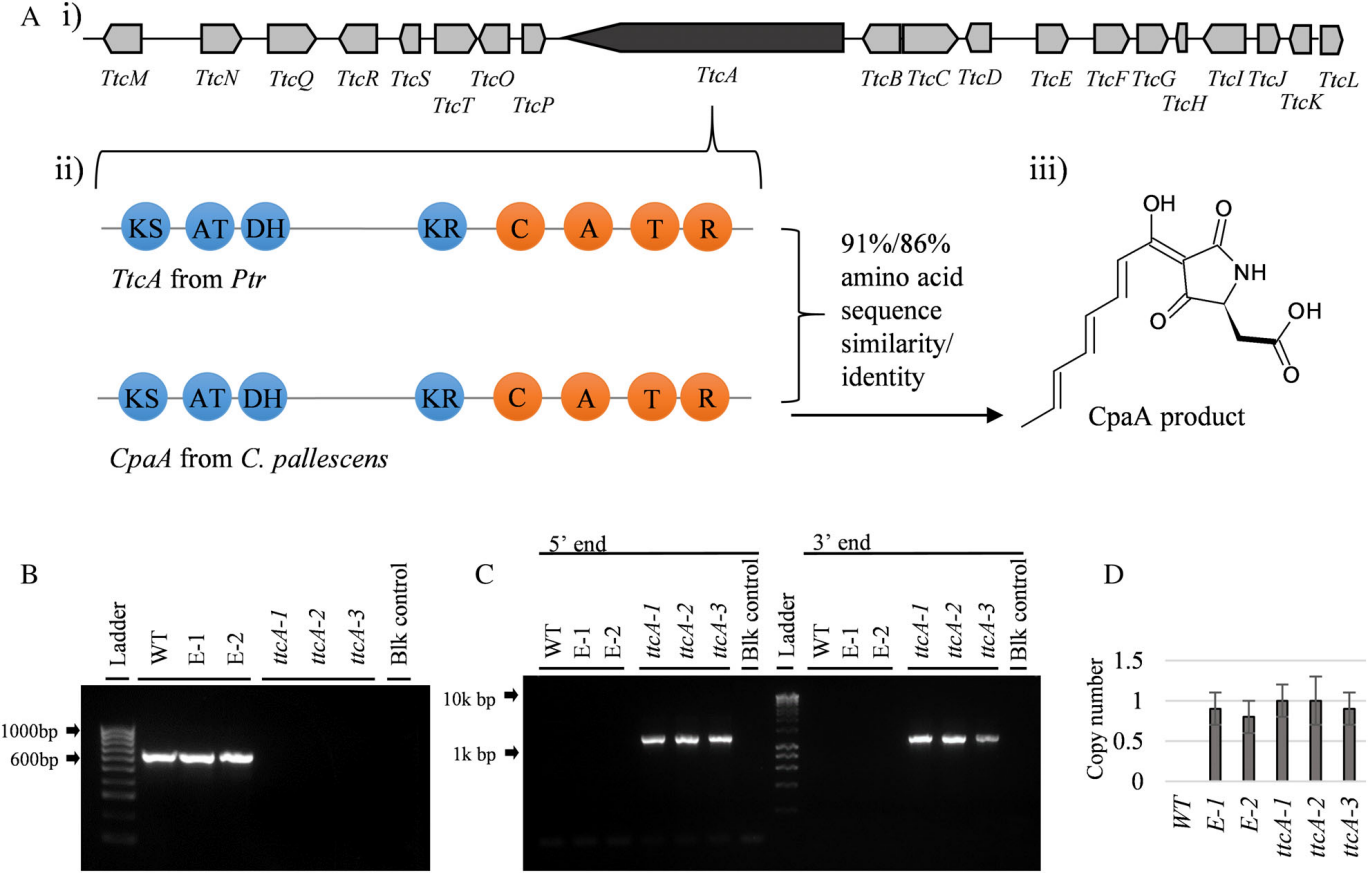
Biosynthesis of triticones. A. i) The *Ttc* biosynthetic genecluster with dark grey representing the core PKS‐NRPS biosynthetic gene and light grey representing ancillary genes ii)the *TtcA* and *CpaA* domain structure, iii) the product of *CpaA*. B. PCR of *TtcA* in WT, ectopic (E‐1 and E‐2) and mutant strains (*ttcA‐1*, *ttcA‐2*, *ttcA‐3*). C. PCR of *TtcA* gene deletion cassette in mutants. D. Phleomycin resistance cassette copy number of mutant and ectopic strains as determined by qPCR. [Color figure can be viewed at http://wileyonlinelibrary.com]

Triticones are spirocyclic γ‐lactams that contain both ketone and amino acid‐derived amide moieties which suggested that they are synthesized by enzymes encoded by hybrid PKS‐NRPS biosynthetic gene clusters. Genome sequence analysis using antiSMASH (FungiSMASH v5.0.0) identified three biosynthetic gene clusters containing both PKS and NRPS genes in M4 (Supporting Information Table S4). Two of the three clusters were discounted as candidates based on the domain structure of the NRPS gene; one (PWO07329) contains a multi‐modular domain structure indicative of multiple amide bond formations and the second has a single incomplete NRPS module (PWO09899) as well as a truncated PKS gene (PWO09906). The third gene cluster, henceforth referred to as *Ttc*, comprised of 20 genes (*TtcA‐L*), is 52 kb in length and contains PtrM4_14012 (*TtcA*) [Fig. 4A(i)], a PKS‐NRPS hybrid with the domain structure KS (ketosynthase), AT (acyltransferase), DH (dehydratase), KR (ketoreductase), C (condensation), A (adenylation), T (thiolation) and R (reductase) [Fig. 4A(ii)]. The TtcA protein has 91%/86% amino acid sequence similarity/identity to the PKS‐NRPS hybrid CpaA found in *C. pallescens*. Comparative analyses between the *Ttc* and *Cpa* biosynthetic gene clusters revealed 123 additional homologues shared by both clusters including *CpaB‐L* (*TtcB‐L*), *CpaO* (*TtcO*) and *CpaP* (*TtcP*) (Supporting Information Table S5 and Fig. S9.).

**Figure 5:**
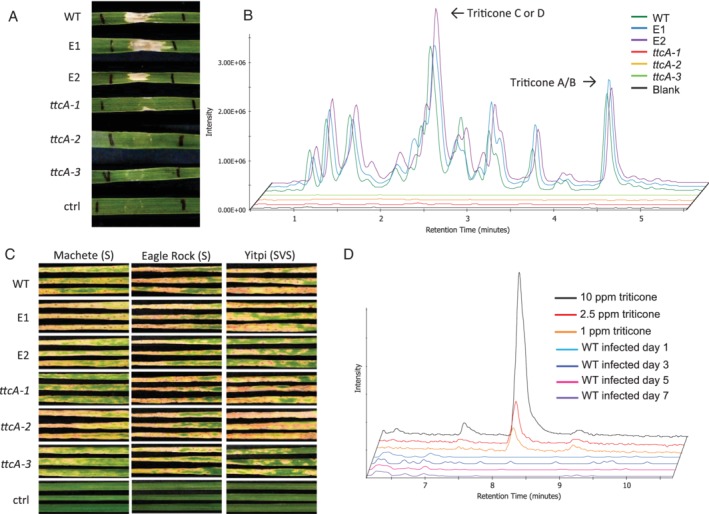
Mutant strains of M4 lacking TtcA. A. Infiltration of crude culture filtrate of M4 (WT), ectopic (E1 and E2), mutant (ttcA‐1, *ttcA‐2*, and *ttcA‐3*) strains and blank control (ctrl) into cv. Eagle Rock. B. Extracted ion chromatogram of amalgamated triticone precursor masses (m/z 278, 280, 280, 298 and 300) in WT, ectopic, mutant strains and blank control. C. Whole plant spray of the WT, ectopic and mutant strains of M4 on to cv. Machete, Eagle Rock and Yitpi. D. Extracted ion chromatogram of m/z 278.1028 (triticone A/B) in plant at day 1, 3, 5 and 7 days post infection compared to a calibration standard of triticone A/B at 1.0, 2.5 and 10 ppm spiked into a non‐infected plant extract. [Color figure can be viewed at http://wileyonlinelibrary.com]

In order to confirm the role of *TtcA* in the production of triticones, the gene was deleted via homologous recombination of a knock‐out construct. Transformed strains were screened via PCR and qPCR (Fig. 4B and C) and three strains (*ttcA‐1*, *ttcA‐2* and *ttcA‐3*) were selected for further study. Two strains (*E‐1* and *E‐2*) containing an ectopic (non‐homologous) insertion of the gene deletion cassette were also selected to confirm integration of the cassette does not induce side effects. The growth and morphology of the *ttcA* mutants and ectopic strains were not significantly affected (Supporting Information Fig. S10). LC‐Q‐MS analysis of the three *ttcA* mutant strains indicated that all 38 triticone compounds were undetectable in the crude culture filtrates and infiltration into wheat produced no necrotic activity (Fig. 5A and B). The ectopic and wild‐type (WT) strains produced culture filtrates with identical levels of triticones and necrotic activity.

### *Triticones do not contribute to pathogenicity during wheat infection*

Seedlings of two tan spot susceptible (S—Eagle Rock and Machete) and one susceptible/very susceptible (SVS—Yitpi) wheat cultivars were infected with spores of the wild type, ectopic and three *ttcA* mutant strains to determine the effect of the deletion on pathogenicity. The mutants retained full pathogenicity and displayed necrotic lesions that were comparable with the wild type and ectopic strains (Fig. 5C). To determine whether triticone A/B was produced by Ptr during infection, Eagle Rock leaves infected with the WT strain were sampled to quantitatively analyse triticone production *in planta* at 1, 3, 5 and 7‐days post‐infection. First and second leaves were harvested, extracted and analysed via high‐resolution LC–MS and the detection limit of triticone A/B in plant material was determined to be 5 μg g^−1^ of lyophilized whole leaf material (Fig. 5D). No triticone candidates were detected in extracts of WT infected leaves at any time point post‐infection (Fig. 5D). The expression of *TtcA* was quantified using qPCR on first and second leaves of WT infected Eagle Rock at 1, 3, 5 and 7‐days post‐infection. No expression of *TtcA* was detected within the first 5 days. Low‐level expression of *TtcA* (0.02 ± 0.00 gene expression relative to actin) was detected at 7 days post‐infection (Supporting Information Fig. S11).

### *Triticone A/B exhibits low‐level antimicrobial activity*

Growth inhibition of fungal and bacterial species by triticone A/B was tested using a disk diffusion method. Disks were infused with 20, 5 and 1 μg triticone A/B and placed on plates of *Pichia pastoris Alternaria infectoria*, *Bipolaris sorokiniana*, *Epicoccum nigrum* and *Parastagonospora nodorum*. No zone of inhibition was observed. Disks were also applied to cultures of the Gram‐negative bacteria, *Escherichia coli* and *Pseudomonas syringae* and Gram‐positive bacteria, *Bacillus subtilis* and *Rhodococcus erythropolis*. The Gram‐negative bacteria were not inhibited, but zones of inhibition of 6.3 mm and 13 mm were observed for *B. subtilis* and *R. erythropolis*, respectively at 20 μg disk^−1^ (Supporting Information Fig. S12).

## Discussion

It has been well established that small molecular weight compounds can contribute to phytopathogenicity in fungal systems (Möbius and Hertweck, 2009; Horbach *et al*., 2011; Pusztahelyi *et al*., 2015) and surveys of the Ptr genome by Manning (2013) and Moolhuijzen (2018), elicited in excess of 20 biosynthetic gene clusters, none of which have been characterized for product or function. Given the prevalence of secondary metabolites involved in pathogenicity, it is not unreasonable to surmise one or more products from these biosynthetic gene clusters may contribute to virulence.

### *The triticones of Ptr*

In pioneering studies, three enantiomeric pairs of triticones were isolated and identified from culture filtrates of Ptr (Fig. 2A) (Sugawara *et al*., 1988; Hallock *et al*., 1993). Triticones A/B, enantiomers at the C‐2 position, were found to be highly phytotoxic. Triticones C/D, also enantiomers at C‐2 but also reduced at C‐4, were found to be mildly phytotoxic. Triticones E/F, with a hydrated methylene group at C‐5′, were reported to be non‐phytotoxic.

We found 38 triticone compounds in Ptr cultures, a quantity exceeding the total number of previously characterized triticones in all fungi. Triticones are known to be unstable during purification and it is possible that some of these compounds are products of rearrangement or degradation (Sugawara *et al*., 1988; Sandmeier and Tamm, 1989a,b; Hallock *et al*., 1993). Sugawara (1988) suggested that the presence of the triticone A/B enantiomer pair may be the product of a retro‐aldol‐type reaction, where cleavage of the carbon bond between C‐1 and C‐2 occurs forming an achiral aldehyde which readily condenses again forming a racemic mixture. Alternatively, all stereoisomers of triticones are potentially produced to varying degrees. For triticone C/D, with three chiral carbons, up to eight stereoisomers are possible; however, 12 compounds were detected with the molecular formula of triticone C/D in the M4 culture filtrate. We found that the number of detectable triticone compounds was highest in strains that produced the most triticone A/B. This is consistent with these other compounds being pathway precursors and minor products or artefacts of rearrangement and degradation.

### *Triticones are produced by the* Ttc *cluster*

The biosynthetic gene cluster *Ttc* was investigated to determine whether it was responsible for synthesis of the triticone class of compounds. The *Ttc* cluster is similar to *Cpa* in *C. pallescens* with strong homology to 14 of the 18 genes within *Cpa*. The *Cpa* cluster synthesizes the curvupallides, fused pyrone/γ‐lactam compounds, structurally similar to the spirocyclic γ‐lactam triticones (Yokoyama *et al*., 2017). Indeed, seven triticones have previously been characterized in *C. pallescens* (Abraham *et al*., 1995a; Abraham *et al*., 1995b) and the product of *CpaA*, a PKS‐NRPS hybrid, has been implicated as the precursor in triticone biosynthesis (Yokoyama *et al*., 2017). As *Ttc* contains all the proposed genes required for curvupallide synthesis, it is possible one or several of the triticone candidates identified via high‐resolution LC–MS/MS analysis may belong to the structurally similar curvupallide class. However, lack of a curvupallide standard prevented identification of curvupallides in Ptr culture filtrate. No phytotoxic activity was observed for the curvupallides when tested against cress seedlings at 100 μg ml^−1^ (Abraham *et al*., 1995b).

### *Bioactivity of triticones*

Triticone A/B gave visible necrotic symptoms on a range of monocot and dicot hosts at 0.5 mg ml^−1^ (1.8 × 10^−3^ M) upon infiltration of attached leaves. Similar results were observed by Masi (2014) using leaf puncture assays on detached leaves on a variety of plant hosts, but Hallock *et al*. (1993) noted necrotic symptoms as low as 10^−5^ M. The greater sensitivity found by Hallock *et al*. (1993) may be due to their use of constant light in contrast to the 12 h photoperiod employed here and by Masi (2014). Fig. 3 indicates that the phytotoxic activity was light dependent.

Six of the Ptr isolates tested produced a high concentration of triticones in the culture filtrate. Given the metabolic cost of producing specialized metabolites, a survival advantage is presumed to be necessary for the retention of the biosynthetic capacity (Calvo *et al*., 2002; Pickens *et al*., 2011). Triticones have been investigated for a wide range of biological activities including anti‐influenza (Wang *et al*., 2018), anti‐leishmanial (de Almeida *et al*., 2017) and phytotoxicity (Sugawara *et al*., 1988; Hallock *et al*., 1993; Masi *et al*., 2014); however, their role in the pathogenicity of Ptr on wheat has never been directly tested. Deletion of *TtcA* in Ptr prevented the production of the all triticone compounds but did not noticeably impact the degree of virulence of M4 on wheat. This was consistent with our observation of very low expression of *TtcA* at 7 days infection. Triticones A/B inhibited growth of *B. subtilis*, a Gram‐positive non‐pathogenic bacterium commonly found in soil and the human gut and *R. erythropolis*, a beneficial plant bacterium. Although further investigation is necessary, triticones may have a role to play in multi‐microbial environments, where competition for resources such as nutrients and space exists. Biosafety regulations mean that virulence assays must be conducted on seedlings in growth chambers which we recognize does not adequately reflect the field environment. It is possible that triticones have a role in pathogenicity on older plants or on pathogen survival between growing seasons. In addition, whilst wheat is the major host of Ptr, it has been detected on rye, oat, barley and a range of non‐cereal grasses (Ali and Francl, 2003; Abdullah *et al*., 2017) and may contribute to virulence on alternate host species.

## Methods and materials

### *Fungal material and culture filtrate production*

Ptr isolate M4, collected in 2009 from Meckering, Western Australia was grown and the culture filtrate was prepared as described previously with some modification (Moffat *et al*., 2014). Briefly, M4 was grown on V8‐PDA plates at room temperature under standard laboratory lighting for 7 days. A 1.4 cm × 2.8 cm area of mycelial growth was excised and homogenized using a mortar and pestle. This was transferred to 480 ml of Fries 3 liquid media in a 2 l Erlenmeyer flask. Liquid cultures were placed in darkness at 27°C, 100 rpm for 3 days and then placed in darkness for 18 days of stationary growth at room temperature. The culture medium was filtered through three layers of milk filter paper (Daviesway, Heidelberg Heights, Victoria, Australia) followed by centrifugation of filtrate at 10 000 rpm for 1 h and then vacuum filtered through a Whatman No. 1 filter paper.

Dialysis of the crude culture filtrate was carried for 48 h using SnakeSkin pleated dialysis tubing (BioRad, Hercules, CA, USA) with a molecular weight cut off of <3.5 kDa in 0.2 M phosphate buffered water (pH 7.4).

### *Toxin purification*

The culture filtrate was first extracted using dispersive solid phase extraction (dSPE). For every 1 l of culture filtrate, 10 g of Bondesil‐C18, 40 μm sorbent (Agilent, Santa Clara, CA, USA) was added to the filtrate and mixed for 1 h at room temperature.

The filtrate and sorbent were vacuum filtered in to a 60 ml reservoir containing a frit to retain only the sorbent and then washed with 2 × 50 ml of water. Adsorbed molecules from the filtrate were eluted from the sorbent by increasing the percentage of methanol in the aqueous elution solvent in 10% increments. Fractions were dried via removal of methanol by rotary evaporation and remaining water was removed by lyophilization. Fractions were resuspended in a known volume of water for bioassay.

The active fraction was further purified using HPLC (Agilent 1200 LC system) coupled to a diode array detector (DAD) for manual fractionation. A quadrupole mass spectrometer with an ESI source was additionally used for profiling fractions. An Agilent C18 column (PrepHT, 150 mm × 21.2 mm × 5 μm) was used for purification and a Phenomenex C18 column (Kinetix, 10 mm × 2.1 mm ID × 2.6 μm; Torrance, CA, USA) was used for profiling fractions. Purification was carried out using 95:5 acetonitrile:water as mobile phase B and 5:95 acetonitrile:water as mobile phase A. The purification gradient started at 20% B and increased to 40% B over 30 min at a flow rate of 6 ml min^−1^. Fractions were manually collected by monitoring 220, 254, 280 and 420 nm on the DAD and infiltrated in to wheat leaves to trace active fractions. For profiling, mobile phase B was 95:5 acetonitrile:water, 0.1% formic acid and mobile phase A was 5:95 acetonitrile:water, 0.1% formic acid. Chromatographic conditions held the column at 0% B for 0.08 min, then increased to 100% over 10 min, %B was then held for a further 3.3 min and then decreased to 0% B over 0.3 min and finally maintained for 2.3 min (16 min total). The flow rate was 0.75 ml min^−1^ and the injection volume was 3 μl. The mass spectrometer was run as full scan (*m/z* 100–1000) in positive ion mode. Drying gas temperature and flow were 350°C and 12 l min^−1^, respectively and the nebulizer pressure was set at 35 psig. Capillary voltage was 3 kV.

### *Toxin characterization*

Crude culture filtrate was analysed with high‐resolution mass spectrometry using the Q Exactive LC–MS (ThermoFisher, Waltham, MA, USA). Briefly, culture filtrate was diluted with water and analysed using a Zorbax Eclipse XDB‐C18 column (RRHD, 100 mm × 2.1 mm ID × 1.8 μm; Agilent). Mobile phase B was acetonitrile with 0.1% formic acid and mobile phase A was water with 0.1% formic acid. Chromatographic conditions held the column at 5% B for 1 min, then increased to 30% over 5 min, %B was further increased to 99% over 5 min and held for 2 min. %B was dropped back down to 5% over 0.1 min and maintained for 1 min (total time 15 min). The injection volume was 2 μl. The mass spectrometer was equipped with an electrospray ionization source (ESI) and set to acquire full scan and data dependant MS/MS at a resolution of 35 000. Scan range for full scan collection was 80–200 m/z and MS/MS spectra were collected at 10, 20 and 40 eV. Spray voltage was set to 3 kV and capillary temperature was 350°C, auxiliary gas flow and temperature were set to 10 l min^−1^ and 350°C respectively. All mass spectral data analysis was carried out using AnalyzerPro v5.7.0.176. NMR analysis was carried out using a Bruker Avance IIIHD 500 MHz. NMR data analysis was carried out using TopSpin v4.0.1.

### *Phytotoxicity and pathogenicity assays*

Infiltration of crude filtrate, processed fungal extracts and infection assays were carried out on wheat (*Triticum aestivum*) cultivars Eagle Rock, Machete, Mace, Magenta and Yitpi, on canola (*Brassica napus*), *Arabidopsis thaliana*, *Nicotiana benthamiana*, *Brachypodium distachyon*, *Lolium perenne* and barley (*Hordeum vulgare*). Seeds were sown in Grade 2 vermiculite (the Perlite and Vermiculite Factory, Jandakot, WA, Australia) in seed trays at 20°C, 12 h day/night cycle in a controlled growth chamber. Wheat, *B. distachyon*, ryegrass and barley were infiltrated upon full expansion of the second leaf. Canola, *A. thaliana* and tobacco were infiltrated upon full expansion of the third leaf. Infiltration was carried out using a 1 ml needleless syringe and the zone was marked with a permanent marker pen. Plants were infiltrated in triplicate and symptoms were evaluated 4 days post infiltration. Symptom intensity was measured as the length of necrotic region compared to the entire infiltration zone and presented as a percentage.

Plant infection assays were performed as previously described (Moffat *et al*., 2014). Briefly, 2‐week old seedlings were sprayed with a 3000 conidia/mL solution in 0.25% gelatin. Disease symptoms were visually assessed and photographed at 7 days post inoculation. Infection experiment was performed twice with three biological replicates.

### *Light sensitivity*

Infiltrated Eagle Rock seedlings were exposed to three different light settings. The first was 12/12 h light/dark cycle and the second was 24 h dark then scored at day 4. The third setting was a 24 h dark period for 4 days followed by 2 days of 12/12 h light/dark cycle and scored immediately after. Triticone A/B was infiltrated in to second leaves of Eagle Rock seedlings at 2 mg ml^−1^ and placed in their respective lighting scenario.

### *Biosynthetic gene cluster prediction and BLASTP search*

The M4 genome (NQIK01000001.1) was mined for biosynthetic gene clusters using antiSMASH v5.0 fungal version (Blin *et al*., 2019). Under ‘analysis options’, ClusterFinder and Use ClusterFinder algorithm for biosynthetic gene cluster (BGC) border prediction were selected. The minimum cluster size in CDS and minimum number of biosynthetic‐related PFAM domains were both set to 5. Minimum ClusterFinder probability was 60%. Under ‘extra features’, all options were selected (KnownClusterBlast, ClusterBlast, SubClusterBlast, smCoG analysis, ActiveSiteFinder, Whole‐genome PFAM analysis and CASSIS). Protein sequences encoded by the *Ttc* cluster were used for BLASTP query against the NCBI GenBank database (https://blast.ncbi.nlm.nih.gov/Blast.cgi).

### *Generation of* TtcA *gene deletion mutant*

The *TtcA* knockout (KO) cassette was constructed using a fusion PCR approach as previously described (Moffat *et al*., 2014) in order to create a *ttcA* mutant strain. Briefly, a phleomycin resistance cassette was amplified from the pAN8‐1 plasmid and fused with flanking regions upstream and downstream of *TtcA* to generate a knock out construct (Supporting Information Fig. S13) (Supporting Information Table S6 for primers). Transformation was carried out using a polyethylene glycol (PEG)‐mediated protoplast method as described by Moffat *et al*. (2014). Screening of three transformants (*ttcA‐1*, *ttcA‐2* and *ttcA‐3*) using PCR was performed to confirm the absence of the target gene (Fig. 4B). Correct insertion of the gene deletion cassette via PCR at the intended homologous site was verified for all mutant strains (*ttcA‐1*, *ttcA‐2* and *ttcA‐3*) and non‐homologous insertion was confirmed for the ectopic strains (*E‐1* and *E‐2*) (Fig. 4C). Confirmation of single‐copy integration for mutants and ectopics was determined by quantitative PCR (qPCR) (Fig. 4D) as described by Moffat *et al*., 2014.

### TtcA *expression analysis during infection*

RNA was extracted from wheat leaf samples infected with WT Ptr at 1, 3, 5 and 7 days post‐inoculation in triplicate. The second leaf was collected and immediately snap‐frozen. Whole leaf material was ground in liquid nitrogen and subjected to RNA extraction using TRIzol reagent and Direct‐zol RNA miniprep kit (Zymo Research, Irvine, CA, USA). Extracted RNA was treated with TURBO DNA‐free kit (Thermo Fischer Scientific, Waltham, MA, USA) and subjected to reverse transcription using the iScript cDNA synthesis kit (Bioline, London, UK) as per manufacturers' protocols. Quantitative RT‐PCR analysis was performed using Quantitect SYBR Green RT‐PCR kit (Qiagen, Valencia, CA, USA) as described by Rybak *et al*. (2017). Primers TritA_F and TritA_R were used to amplify the 150 bp region of *TtcA* (Supporting Information Table S6 for primers). To normalize the gene expression, primer set Act1F4 and Act1R4 were used to amplify the housekeeping gene actin. Samples were analysed with three biological replicates and two‐technical replicates.

### In planta *detection of triticones using LC–MS*

Wheat seedlings (Eagle Rock) with fully expanded third leaves were inoculated with WT, ectopic and *ttcA* strains. Plant infection assays were performed as described above but with three biological replicates. A lower conidial concentration of 1000 conidia/mL was used for *ttcA*‐3 due to insufficient spore production. The second leaf of infected seedlings was collected, snap frozen and freeze‐dried at 1, 3, 5 and 7 days post inoculation. Leaves were ground to a fine powder and 10 mg was extracted with 2 × 500 μl methanol and 1 × 500 μl 50:50 water:methanol. Combined extracts were split to represent 5 mg extractions, solvents were removed and reconstituted in 100 μl 95:5 water:acetonitrile and analysed via high‐resolution LC–MS as described in Toxin Characterization.

### *Antimicrobial assays*

Antimicrobial activity of triticone A/B was assessed in fungal and bacterial species using a disk diffusion method (Bauer *et al*., 1966). Antifungal activity for triticone A/B was tested against *Pichia pastoris*, A*lternaria infectoria*, *Bipolaris sorokiniana*, *Epicoccum nigrum* and *Parastagonospora nodorum*. Briefly, for filamentous fungi 50 μl of spore suspensions were each spread on V8‐PDA plates and 10 μl of triticone A/B solution at 2.0, 0.5 and 0.1 mg ml^−1^ was applied to 5 mm filter paper disks placed on the inoculated plates (equivalent to 20 μg/disc). Plates were grown at room temperature for 36 to 72 h and plates were observed for any growth inhibition. *P. pastoris* was cultured to an optical density (OD) of approximately 0.6–0.8 and 100 μl was spread on YPD plates and grown at 28°C.

Antibacterial activity was similarly tested against two Gram‐negative bacteria *Escherichia coli* and *Pseudomonas syringae* and two Gram‐positive bacteria *Bacillus subtilis* and *Rhodoccus erythropolis* strain 66b. Briefly, bacterial suspensions were grown to approximately 0.6–0.8 OD at 37°C and 100 μl was spread and dried over LB agar plates. Filter paper disks were infused with 10 μl of triticone A/B solution at 2, 0.5 and 0.1 mg ml^−1^ and the inhibition zone was measured in millimetres, 24 h after inoculation.

## Supporting information


**Appendix S1:** Supporting Information

**Appendix S2:** Supporting Information
