# Supplementary material for: The identification and deletion of the polyketide synthase‐nonribosomal peptide synthase gene responsible for the production of the phytotoxic triticone A/B in the wheat fungal pathogen Pyrenophora tritici‐repentis
Source: Environ Microbiol. 2019 Nov 21;21(12):4875–86. doi: 10.1111/1462-2920.14854 (PMC6915911; doi:10.1111/1462-2920.14854)
Supplement: Supplementary file 1 — Appendix S1: Supporting Information [file EMI-21-4875-s001.pptx]

## Slide 1
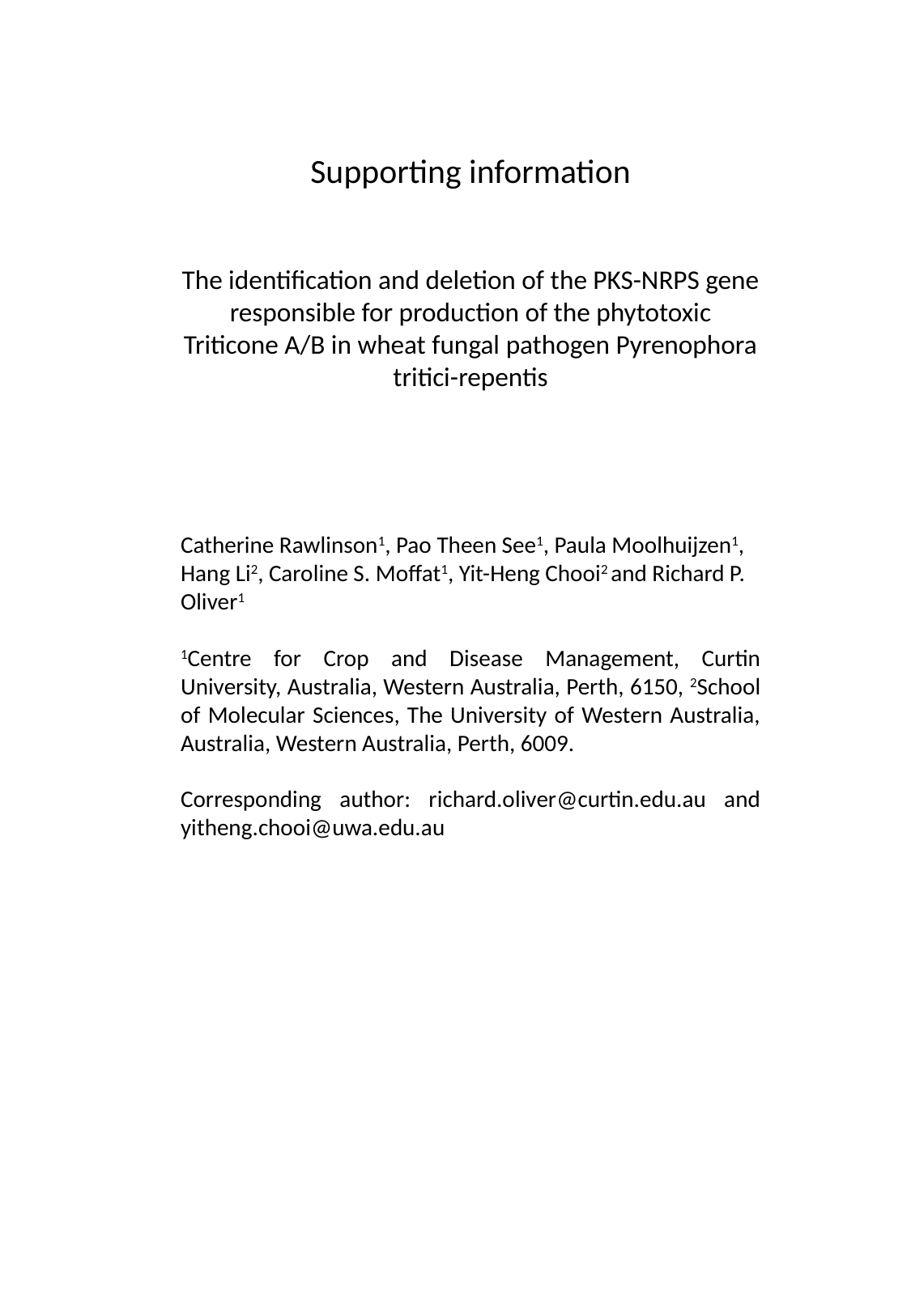

Supporting information
The identification and deletion of the PKS-NRPS gene responsible for production of the phytotoxic Triticone A/B in wheat fungal pathogen Pyrenophora tritici-repentis
Catherine Rawlinson1, Pao Theen See1, Paula Moolhuijzen1, Hang Li2, Caroline S. Moffat1, Yit-Heng Chooi2 and Richard P. Oliver1
1Centre for Crop and Disease Management, Curtin University, Australia, Western Australia, Perth, 6150, 2School of Molecular Sciences, The University of Western Australia, Australia, Western Australia, Perth, 6009.
Corresponding author: richard.oliver@curtin.edu.au and yitheng.chooi@uwa.edu.au

## Slide 2
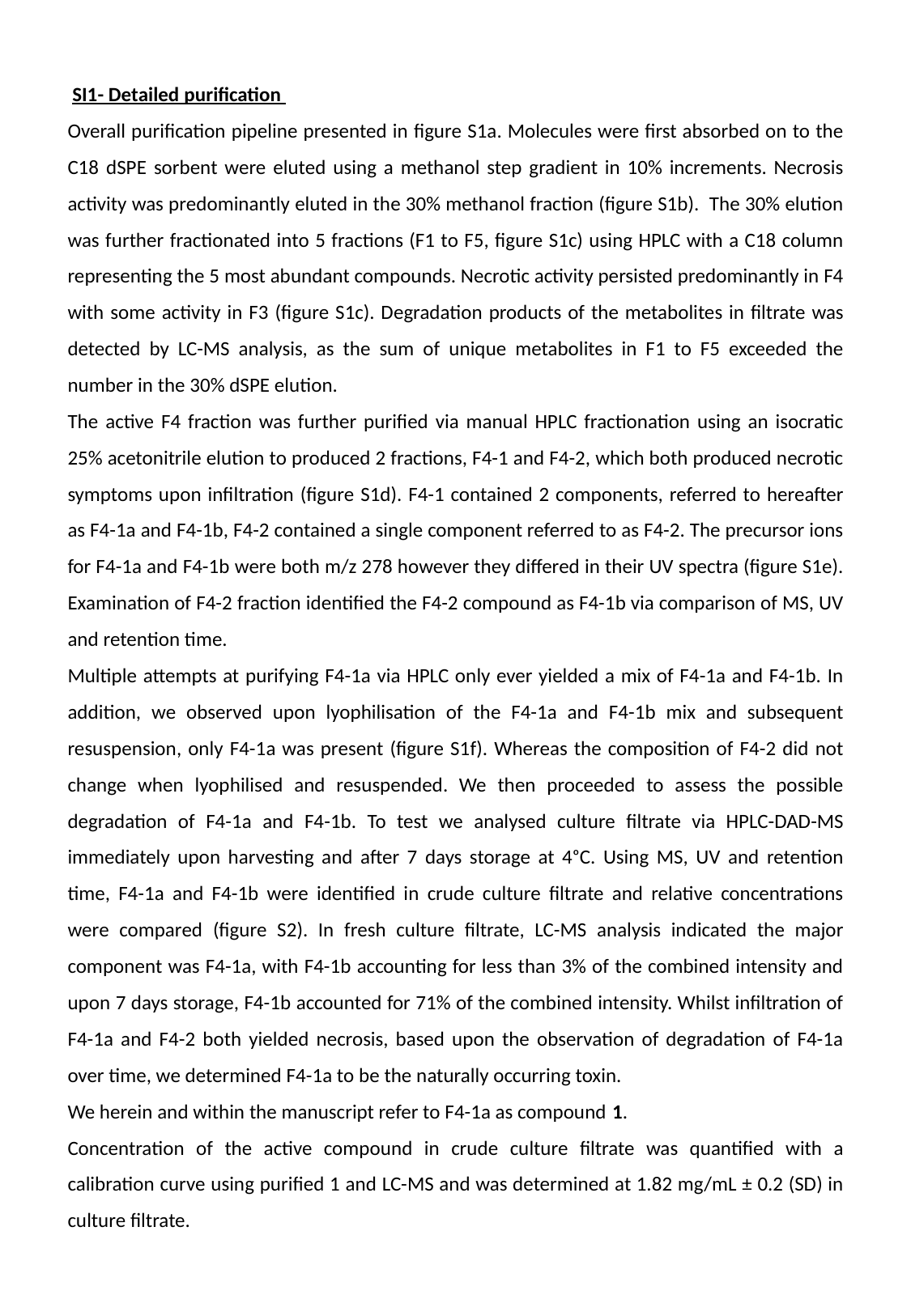

SI1- Detailed purification
Overall purification pipeline presented in figure S1a. Molecules were first absorbed on to the C18 dSPE sorbent were eluted using a methanol step gradient in 10% increments. Necrosis activity was predominantly eluted in the 30% methanol fraction (figure S1b). The 30% elution was further fractionated into 5 fractions (F1 to F5, figure S1c) using HPLC with a C18 column representing the 5 most abundant compounds. Necrotic activity persisted predominantly in F4 with some activity in F3 (figure S1c). Degradation products of the metabolites in filtrate was detected by LC-MS analysis, as the sum of unique metabolites in F1 to F5 exceeded the number in the 30% dSPE elution.
The active F4 fraction was further purified via manual HPLC fractionation using an isocratic 25% acetonitrile elution to produced 2 fractions, F4-1 and F4-2, which both produced necrotic symptoms upon infiltration (figure S1d). F4-1 contained 2 components, referred to hereafter as F4-1a and F4-1b, F4-2 contained a single component referred to as F4-2. The precursor ions for F4-1a and F4-1b were both m/z 278 however they differed in their UV spectra (figure S1e). Examination of F4-2 fraction identified the F4-2 compound as F4-1b via comparison of MS, UV and retention time.
Multiple attempts at purifying F4-1a via HPLC only ever yielded a mix of F4-1a and F4-1b. In addition, we observed upon lyophilisation of the F4-1a and F4-1b mix and subsequent resuspension, only F4-1a was present (figure S1f). Whereas the composition of F4-2 did not change when lyophilised and resuspended. We then proceeded to assess the possible degradation of F4-1a and F4-1b. To test we analysed culture filtrate via HPLC-DAD-MS immediately upon harvesting and after 7 days storage at 4ᵒC. Using MS, UV and retention time, F4-1a and F4-1b were identified in crude culture filtrate and relative concentrations were compared (figure S2). In fresh culture filtrate, LC-MS analysis indicated the major component was F4-1a, with F4-1b accounting for less than 3% of the combined intensity and upon 7 days storage, F4-1b accounted for 71% of the combined intensity. Whilst infiltration of F4-1a and F4-2 both yielded necrosis, based upon the observation of degradation of F4-1a over time, we determined F4-1a to be the naturally occurring toxin.
We herein and within the manuscript refer to F4-1a as compound 1.
Concentration of the active compound in crude culture filtrate was quantified with a calibration curve using purified 1 and LC-MS and was determined at 1.82 mg/mL ± 0.2 (SD) in culture filtrate.

## Slide 3
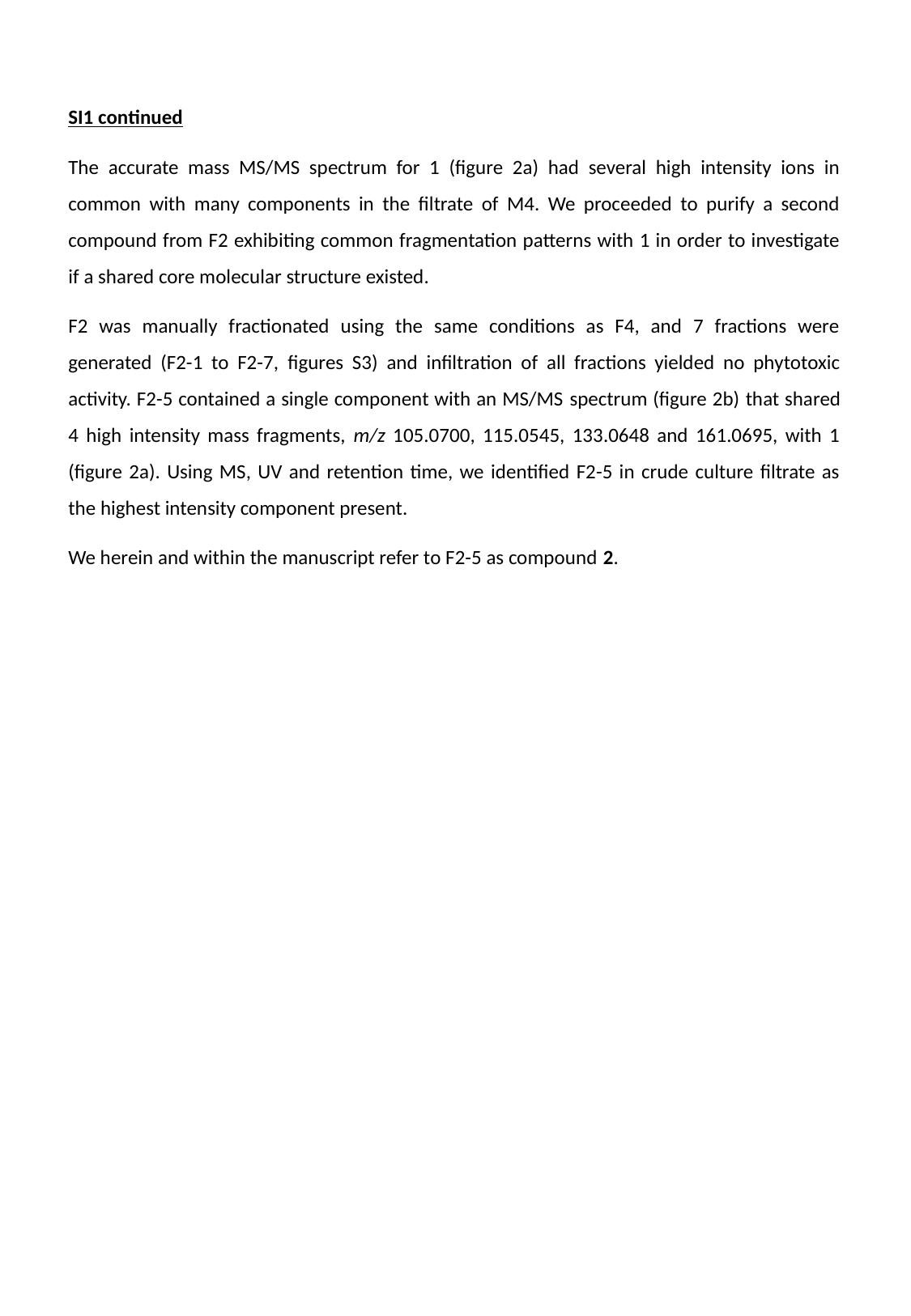

SI1 continued
The accurate mass MS/MS spectrum for 1 (figure 2a) had several high intensity ions in common with many components in the filtrate of M4. We proceeded to purify a second compound from F2 exhibiting common fragmentation patterns with 1 in order to investigate if a shared core molecular structure existed.
F2 was manually fractionated using the same conditions as F4, and 7 fractions were generated (F2-1 to F2-7, figures S3) and infiltration of all fractions yielded no phytotoxic activity. F2-5 contained a single component with an MS/MS spectrum (figure 2b) that shared 4 high intensity mass fragments, m/z 105.0700, 115.0545, 133.0648 and 161.0695, with 1 (figure 2a). Using MS, UV and retention time, we identified F2-5 in crude culture filtrate as the highest intensity component present.
We herein and within the manuscript refer to F2-5 as compound 2.

## Slide 4
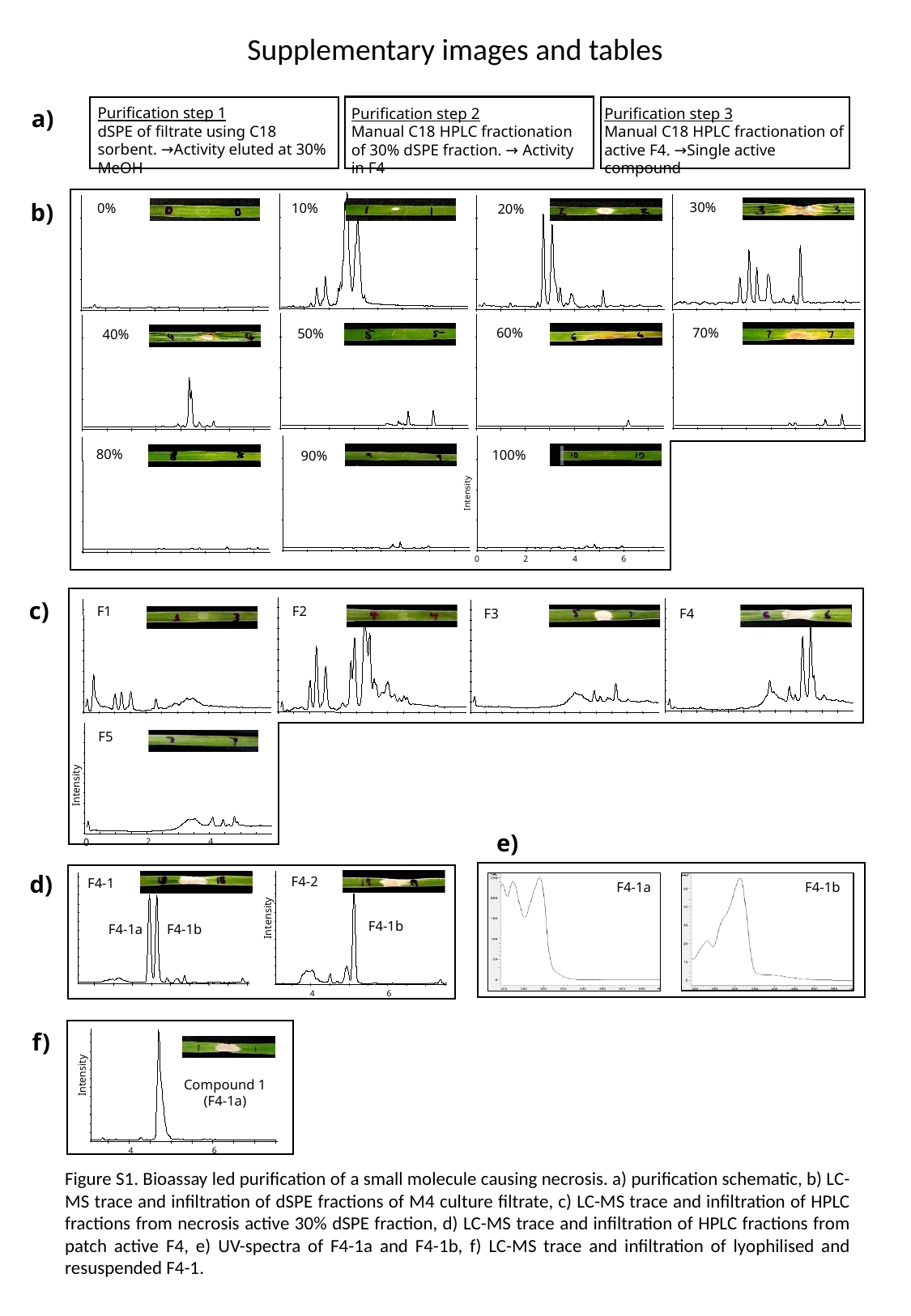

Supplementary images and tables
Purification step 2
Manual C18 HPLC fractionation of 30% dSPE fraction. → Activity in F4
Purification step 3
Manual C18 HPLC fractionation of active F4. →Single active compound
Purification step 1
dSPE of filtrate using C18 sorbent. →Activity eluted at 30% MeOH
a)
b)
10%
30%
0%
20%
50%
70%
60%
40%
90%
Intensity
0
2
4
6
100%
80%
F1
F2
F4
F3
F5
Intensity
0
2
4
c)
e)
d)
F4-2
Intensity
4
6
F4-1b
F4-1
F4-1a
F4-1b
F4-1a
F4-1b
Intensity
4
6
Compound 1
(F4-1a)
f)
Figure S1. Bioassay led purification of a small molecule causing necrosis. a) purification schematic, b) LC-MS trace and infiltration of dSPE fractions of M4 culture filtrate, c) LC-MS trace and infiltration of HPLC fractions from necrosis active 30% dSPE fraction, d) LC-MS trace and infiltration of HPLC fractions from patch active F4, e) UV-spectra of F4-1a and F4-1b, f) LC-MS trace and infiltration of lyophilised and resuspended F4-1.

## Slide 5
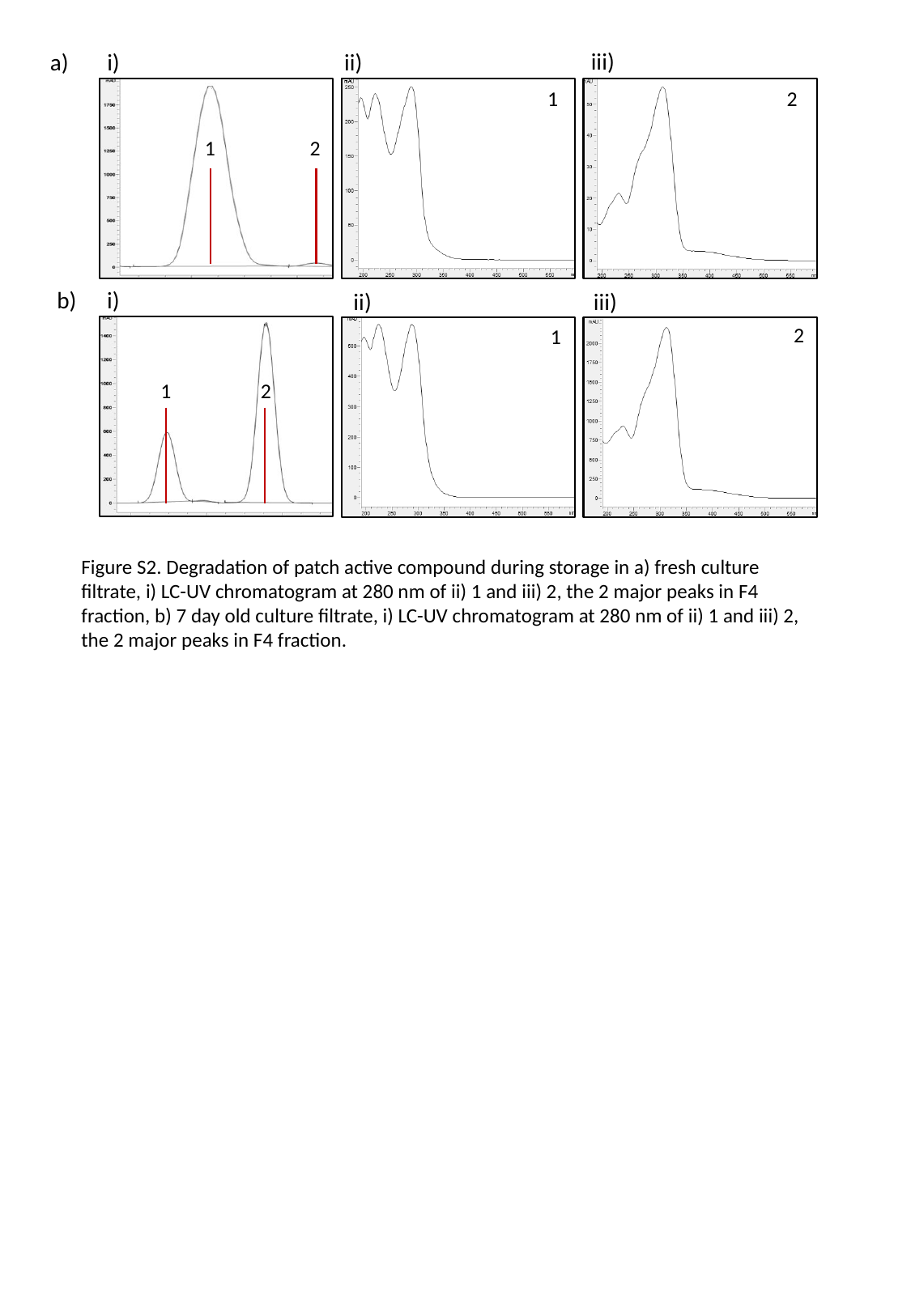

iii)
a)
ii)
1
2
2
1
b)
iii)
ii)
2
1
1
2
i)
i)
Figure S2. Degradation of patch active compound during storage in a) fresh culture filtrate, i) LC-UV chromatogram at 280 nm of ii) 1 and iii) 2, the 2 major peaks in F4 fraction, b) 7 day old culture filtrate, i) LC-UV chromatogram at 280 nm of ii) 1 and iii) 2, the 2 major peaks in F4 fraction.

## Slide 6
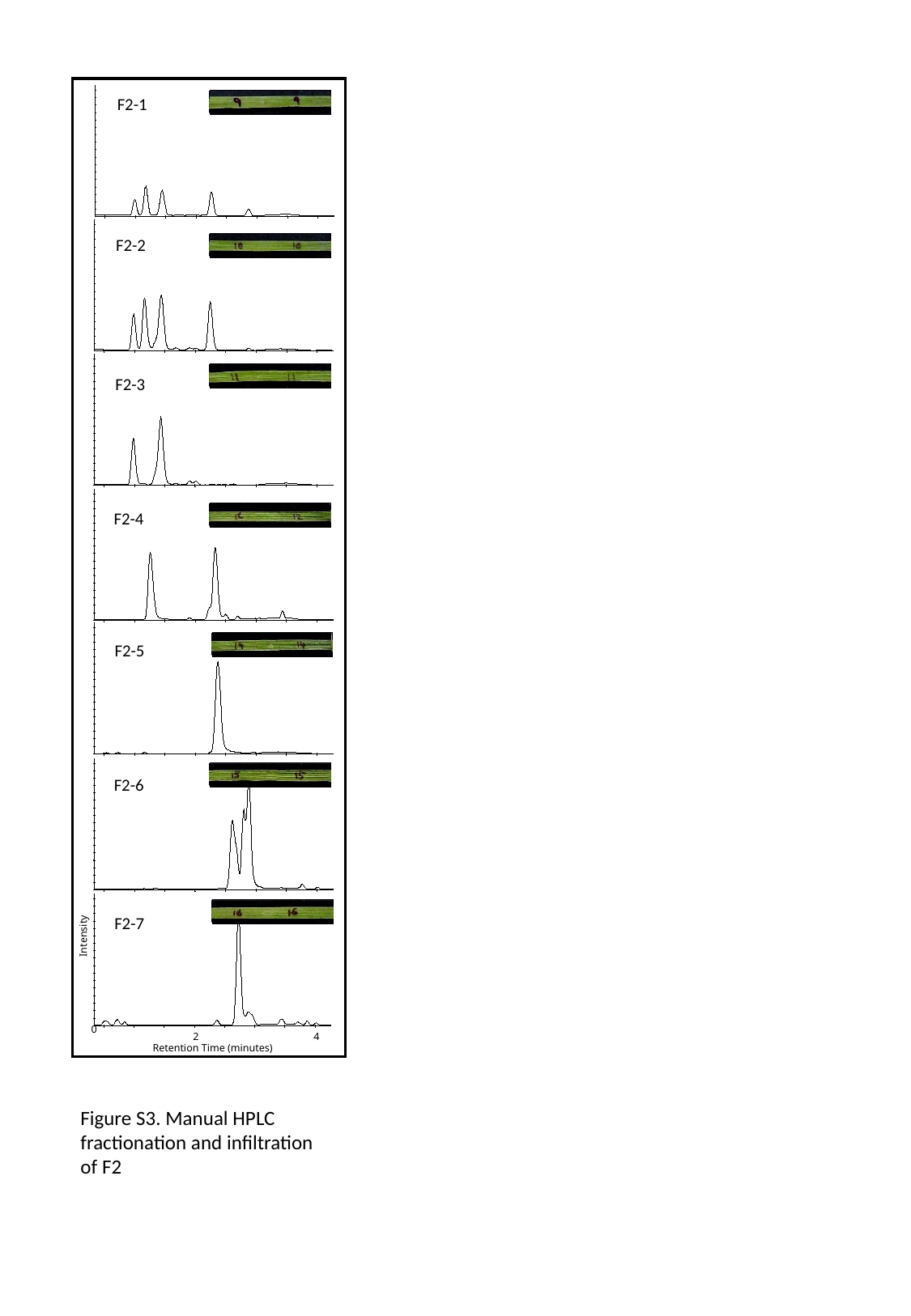

F2-1
F2-2
F2-3
F2-4
F2-5
F2-6
0
2
4
Retention Time (minutes)
F2-7
Intensity
Figure S3. Manual HPLC fractionation and infiltration of F2

## Slide 7
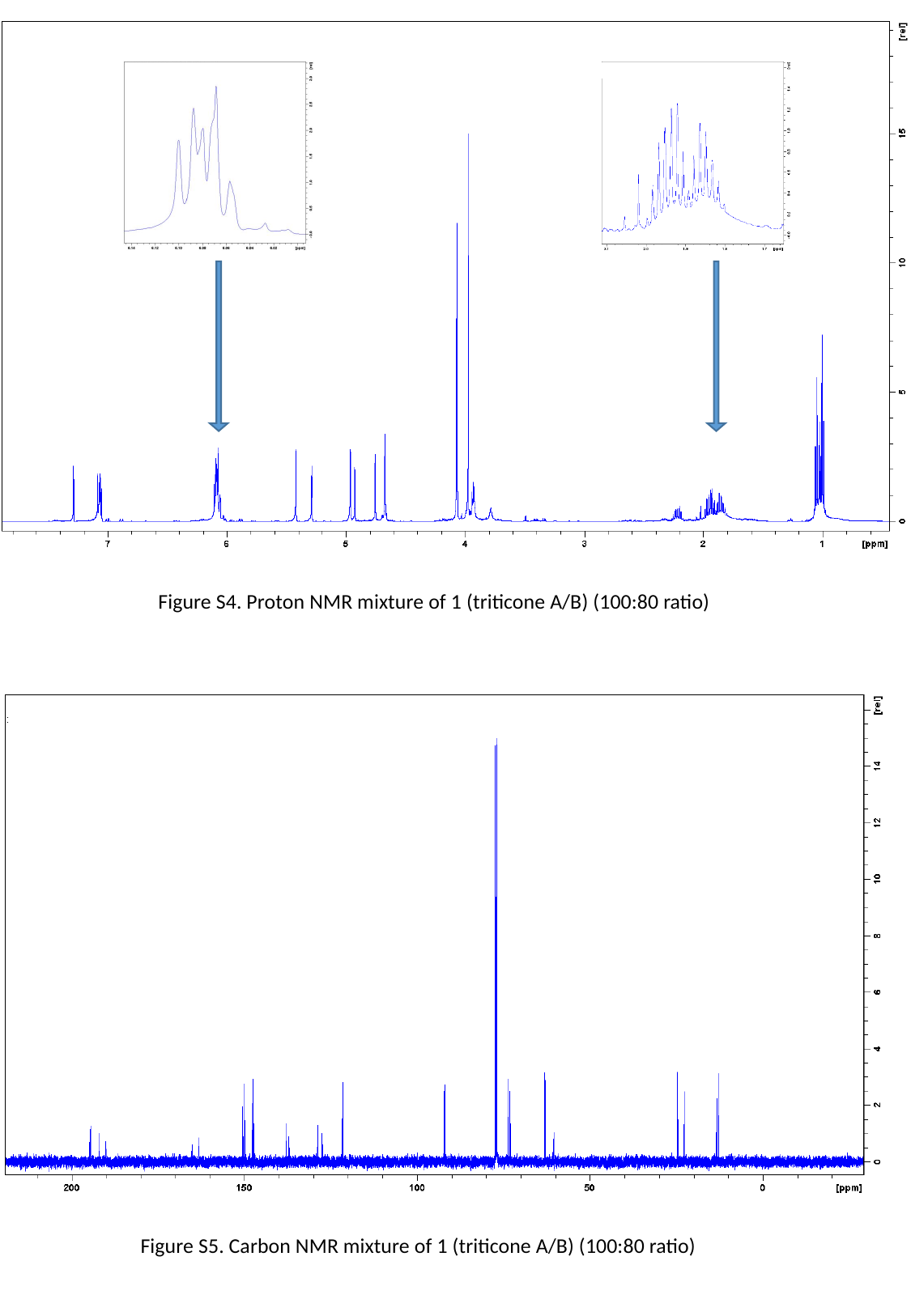

Figure S4. Proton NMR mixture of 1 (triticone A/B) (100:80 ratio)
Figure S5. Carbon NMR mixture of 1 (triticone A/B) (100:80 ratio)

## Slide 8
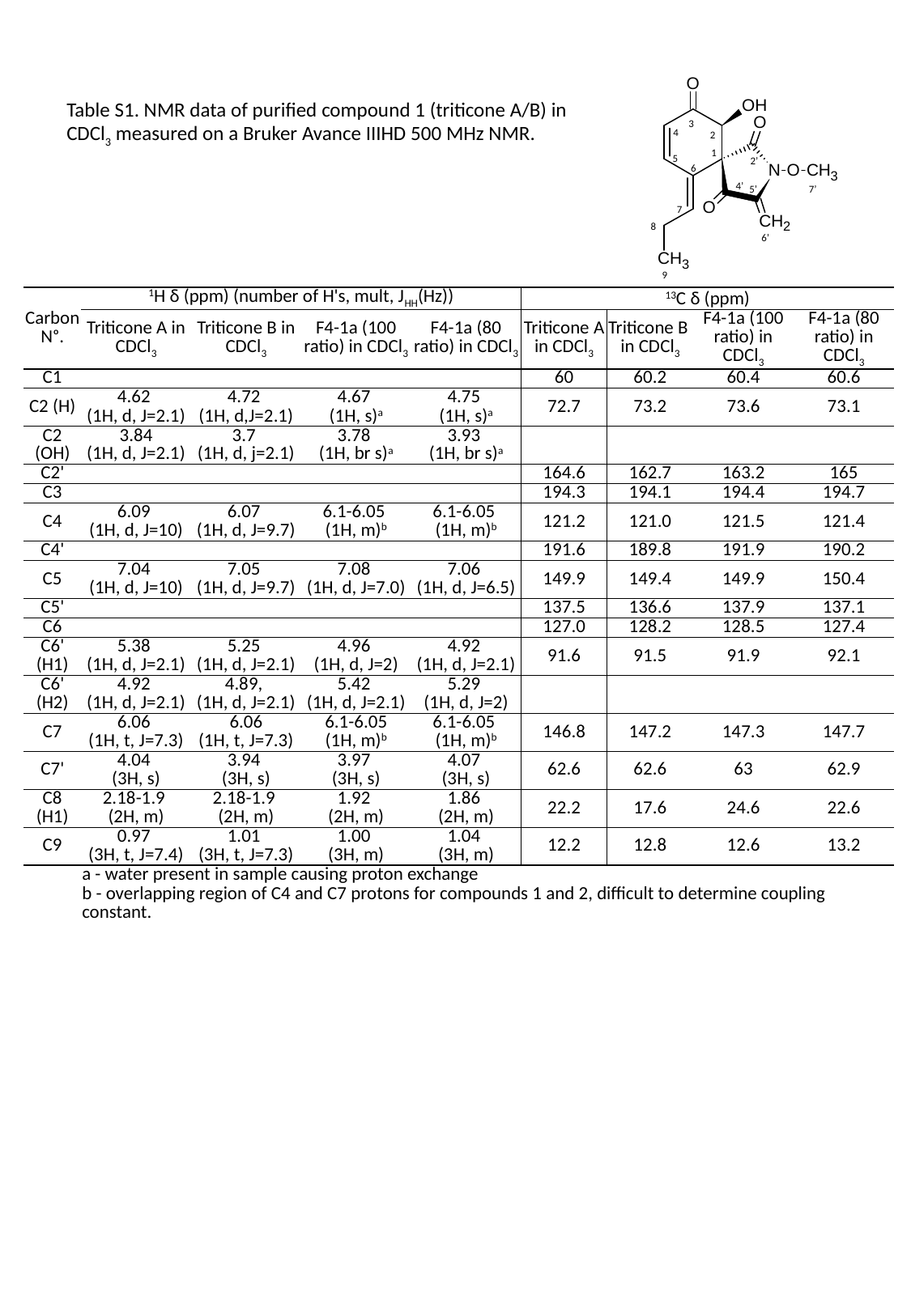

3
4
2
1
5
2’
6
4’
5’
7’
7
8
6’
9
Table S1. NMR data of purified compound 1 (triticone A/B) in CDCl3 measured on a Bruker Avance IIIHD 500 MHz NMR.
| Carbon Nᵒ. | 1H δ (ppm) (number of H's, mult, JHH(Hz)) | | | | 13C δ (ppm) | | | |
| --- | --- | --- | --- | --- | --- | --- | --- | --- |
| | Triticone A in CDCl3 | Triticone B in CDCl3 | F4-1a (100 ratio) in CDCl3 | F4-1a (80 ratio) in CDCl3 | Triticone A in CDCl3 | Triticone B in CDCl3 | F4-1a (100 ratio) in CDCl3 | F4-1a (80 ratio) in CDCl3 |
| C1 | | | | | 60 | 60.2 | 60.4 | 60.6 |
| C2 (H) | 4.62 (1H, d, J=2.1) | 4.72 (1H, d,J=2.1) | 4.67 (1H, s)a | 4.75 (1H, s)a | 72.7 | 73.2 | 73.6 | 73.1 |
| C2 (OH) | 3.84 (1H, d, J=2.1) | 3.7 (1H, d, j=2.1) | 3.78 (1H, br s)a | 3.93 (1H, br s)a | | | | |
| C2' | | | | | 164.6 | 162.7 | 163.2 | 165 |
| C3 | | | | | 194.3 | 194.1 | 194.4 | 194.7 |
| C4 | 6.09 (1H, d, J=10) | 6.07 (1H, d, J=9.7) | 6.1-6.05 (1H, m)b | 6.1-6.05 (1H, m)b | 121.2 | 121.0 | 121.5 | 121.4 |
| C4' | | | | | 191.6 | 189.8 | 191.9 | 190.2 |
| C5 | 7.04 (1H, d, J=10) | 7.05 (1H, d, J=9.7) | 7.08 (1H, d, J=7.0) | 7.06 (1H, d, J=6.5) | 149.9 | 149.4 | 149.9 | 150.4 |
| C5' | | | | | 137.5 | 136.6 | 137.9 | 137.1 |
| C6 | | | | | 127.0 | 128.2 | 128.5 | 127.4 |
| C6' (H1) | 5.38 (1H, d, J=2.1) | 5.25 (1H, d, J=2.1) | 4.96 (1H, d, J=2) | 4.92 (1H, d, J=2.1) | 91.6 | 91.5 | 91.9 | 92.1 |
| C6' (H2) | 4.92 (1H, d, J=2.1) | 4.89, (1H, d, J=2.1) | 5.42 (1H, d, J=2.1) | 5.29 (1H, d, J=2) | | | | |
| C7 | 6.06 (1H, t, J=7.3) | 6.06 (1H, t, J=7.3) | 6.1-6.05 (1H, m)b | 6.1-6.05 (1H, m)b | 146.8 | 147.2 | 147.3 | 147.7 |
| C7' | 4.04 (3H, s) | 3.94 (3H, s) | 3.97 (3H, s) | 4.07 (3H, s) | 62.6 | 62.6 | 63 | 62.9 |
| C8 (H1) | 2.18-1.9 (2H, m) | 2.18-1.9 (2H, m) | 1.92 (2H, m) | 1.86 (2H, m) | 22.2 | 17.6 | 24.6 | 22.6 |
| C9 | 0.97 (3H, t, J=7.4) | 1.01 (3H, t, J=7.3) | 1.00 (3H, m) | 1.04 (3H, m) | 12.2 | 12.8 | 12.6 | 13.2 |
| | a - water present in sample causing proton exchange | | | | | | | |
| | b - overlapping region of C4 and C7 protons for compounds 1 and 2, difficult to determine coupling constant. | | | | | | | |

## Slide 9
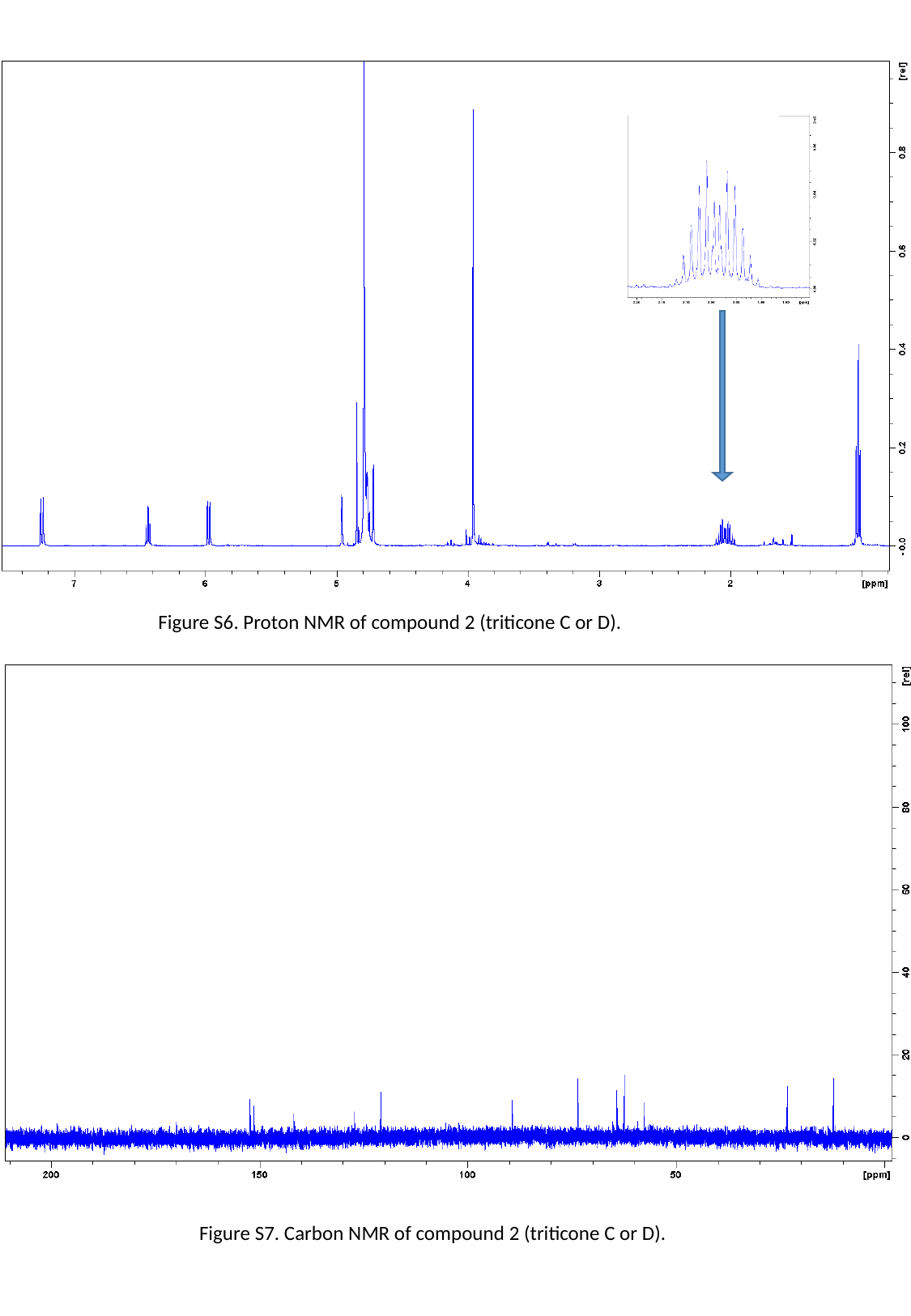

Figure S6. Proton NMR of compound 2 (triticone C or D).
Figure S7. Carbon NMR of compound 2 (triticone C or D).

## Slide 10
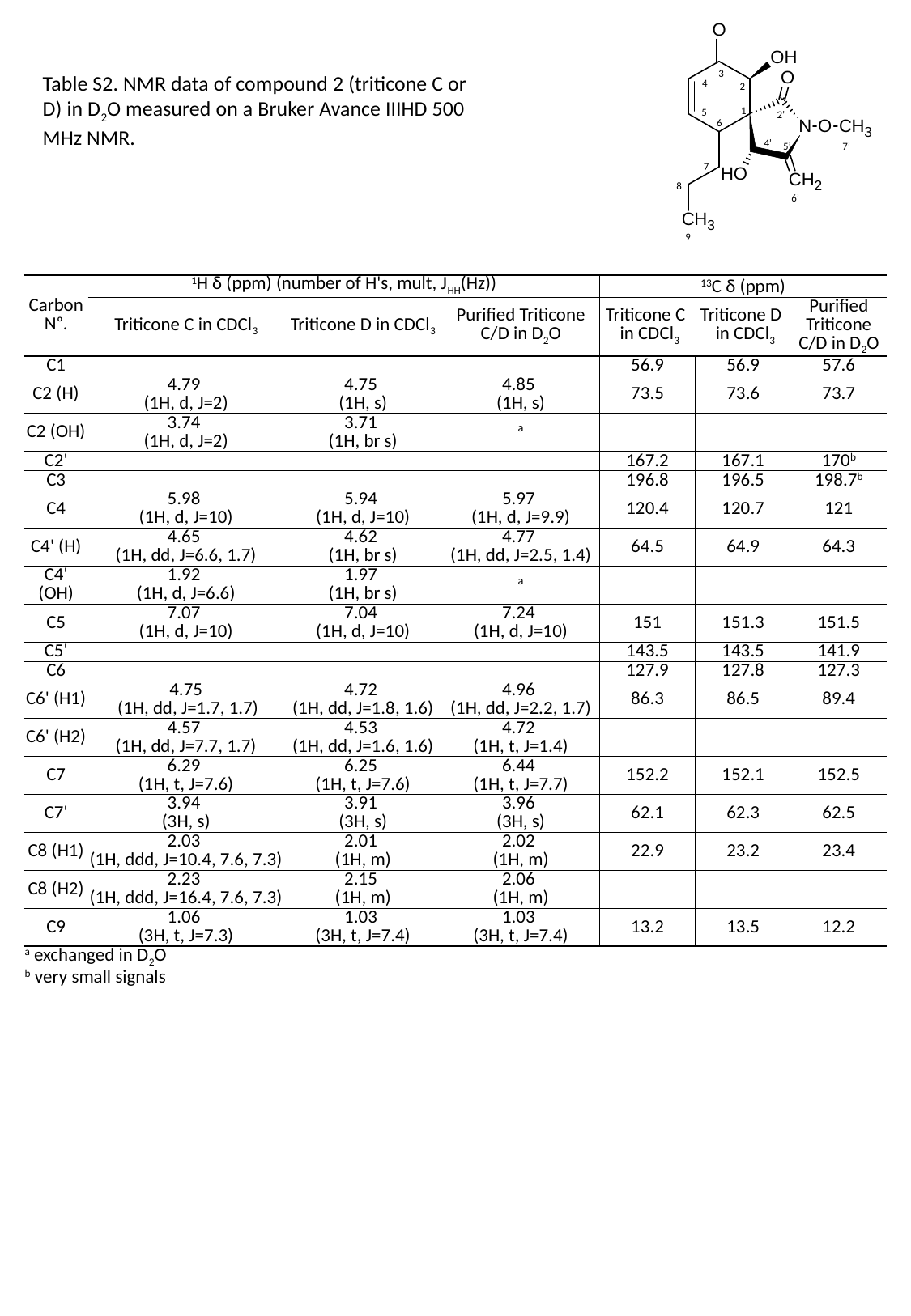

3
4
2
1
5
2’
6
4’
7’
5’
7
8
6’
9
Table S2. NMR data of compound 2 (triticone C or D) in D2O measured on a Bruker Avance IIIHD 500 MHz NMR.
| Carbon Nᵒ. | 1H δ (ppm) (number of H's, mult, JHH(Hz)) | | | 13C δ (ppm) | | |
| --- | --- | --- | --- | --- | --- | --- |
| | Triticone C in CDCl3 | Triticone D in CDCl3 | Purified Triticone C/D in D2O | Triticone C in CDCl3 | Triticone D in CDCl3 | Purified Triticone C/D in D2O |
| C1 | | | | 56.9 | 56.9 | 57.6 |
| C2 (H) | 4.79 (1H, d, J=2) | 4.75 (1H, s) | 4.85 (1H, s) | 73.5 | 73.6 | 73.7 |
| C2 (OH) | 3.74 (1H, d, J=2) | 3.71 (1H, br s) | a | | | |
| C2' | | | | 167.2 | 167.1 | 170b |
| C3 | | | | 196.8 | 196.5 | 198.7b |
| C4 | 5.98 (1H, d, J=10) | 5.94 (1H, d, J=10) | 5.97 (1H, d, J=9.9) | 120.4 | 120.7 | 121 |
| C4' (H) | 4.65 (1H, dd, J=6.6, 1.7) | 4.62 (1H, br s) | 4.77 (1H, dd, J=2.5, 1.4) | 64.5 | 64.9 | 64.3 |
| C4' (OH) | 1.92 (1H, d, J=6.6) | 1.97 (1H, br s) | a | | | |
| C5 | 7.07 (1H, d, J=10) | 7.04 (1H, d, J=10) | 7.24 (1H, d, J=10) | 151 | 151.3 | 151.5 |
| C5' | | | | 143.5 | 143.5 | 141.9 |
| C6 | | | | 127.9 | 127.8 | 127.3 |
| C6' (H1) | 4.75 (1H, dd, J=1.7, 1.7) | 4.72 (1H, dd, J=1.8, 1.6) | 4.96 (1H, dd, J=2.2, 1.7) | 86.3 | 86.5 | 89.4 |
| C6' (H2) | 4.57 (1H, dd, J=7.7, 1.7) | 4.53 (1H, dd, J=1.6, 1.6) | 4.72 (1H, t, J=1.4) | | | |
| C7 | 6.29 (1H, t, J=7.6) | 6.25 (1H, t, J=7.6) | 6.44 (1H, t, J=7.7) | 152.2 | 152.1 | 152.5 |
| C7' | 3.94 (3H, s) | 3.91 (3H, s) | 3.96 (3H, s) | 62.1 | 62.3 | 62.5 |
| C8 (H1) | 2.03 (1H, ddd, J=10.4, 7.6, 7.3) | 2.01 (1H, m) | 2.02 (1H, m) | 22.9 | 23.2 | 23.4 |
| C8 (H2) | 2.23 (1H, ddd, J=16.4, 7.6, 7.3) | 2.15 (1H, m) | 2.06 (1H, m) | | | |
| C9 | 1.06 (3H, t, J=7.3) | 1.03 (3H, t, J=7.4) | 1.03 (3H, t, J=7.4) | 13.2 | 13.5 | 12.2 |
| a exchanged in D2O | | | | | | |
| b very small signals | | | | | | |

## Slide 11
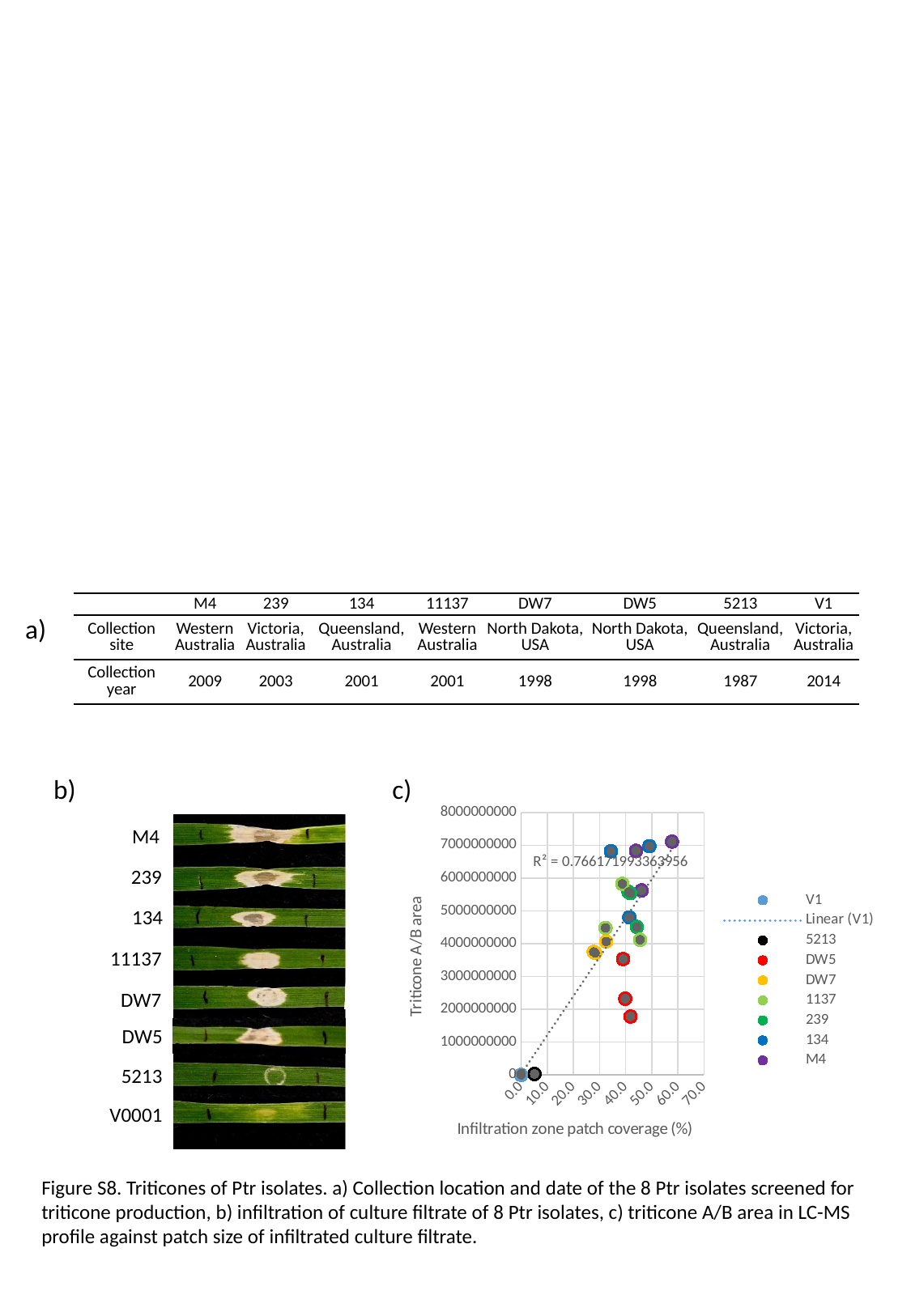

| | M4 | 239 | 134 | 11137 | DW7 | DW5 | 5213 | V1 |
| --- | --- | --- | --- | --- | --- | --- | --- | --- |
| Collection site | Western Australia | Victoria, Australia | Queensland, Australia | Western Australia | North Dakota, USA | North Dakota, USA | Queensland, Australia | Victoria, Australia |
| Collection year | 2009 | 2003 | 2001 | 2001 | 1998 | 1998 | 1987 | 2014 |
a)
b)
c)
### Chart
| Category | | | | | | | | | |
|---|---|---|---|---|---|---|---|---|---|
M4
239
134
11137
DW7
DW5
5213
V0001
Figure S8. Triticones of Ptr isolates. a) Collection location and date of the 8 Ptr isolates screened for triticone production, b) infiltration of culture filtrate of 8 Ptr isolates, c) triticone A/B area in LC-MS profile against patch size of infiltrated culture filtrate.

## Slide 12
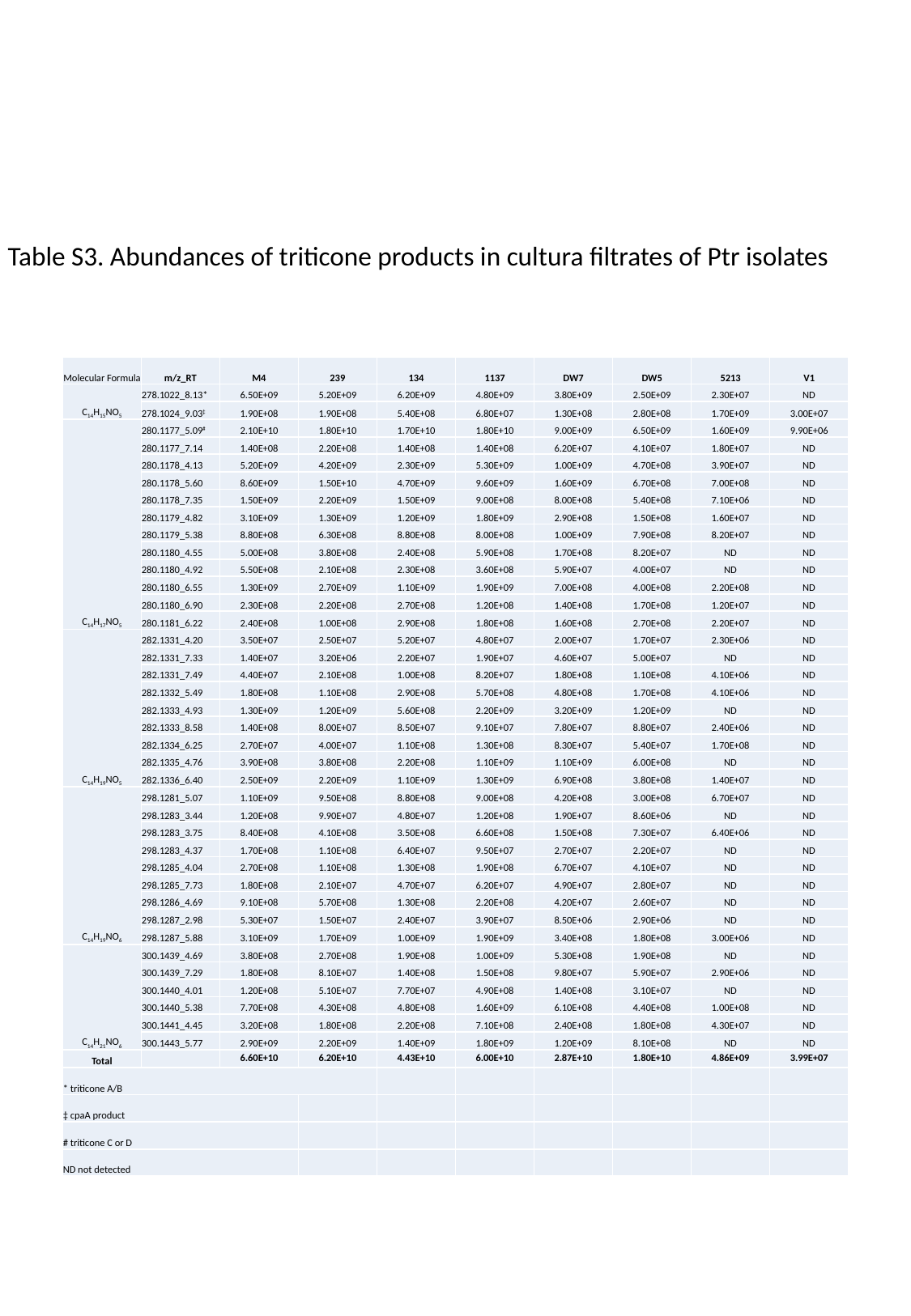

Table S3. Abundances of triticone products in cultura filtrates of Ptr isolates
| Molecular Formula | m/z\_RT | M4 | 239 | 134 | 1137 | DW7 | DW5 | 5213 | V1 |
| --- | --- | --- | --- | --- | --- | --- | --- | --- | --- |
| C14H15NO5 | 278.1022\_8.13\* | 6.50E+09 | 5.20E+09 | 6.20E+09 | 4.80E+09 | 3.80E+09 | 2.50E+09 | 2.30E+07 | ND |
| | 278.1024\_9.03‡ | 1.90E+08 | 1.90E+08 | 5.40E+08 | 6.80E+07 | 1.30E+08 | 2.80E+08 | 1.70E+09 | 3.00E+07 |
| C14H17NO5 | 280.1177\_5.09# | 2.10E+10 | 1.80E+10 | 1.70E+10 | 1.80E+10 | 9.00E+09 | 6.50E+09 | 1.60E+09 | 9.90E+06 |
| | 280.1177\_7.14 | 1.40E+08 | 2.20E+08 | 1.40E+08 | 1.40E+08 | 6.20E+07 | 4.10E+07 | 1.80E+07 | ND |
| | 280.1178\_4.13 | 5.20E+09 | 4.20E+09 | 2.30E+09 | 5.30E+09 | 1.00E+09 | 4.70E+08 | 3.90E+07 | ND |
| | 280.1178\_5.60 | 8.60E+09 | 1.50E+10 | 4.70E+09 | 9.60E+09 | 1.60E+09 | 6.70E+08 | 7.00E+08 | ND |
| | 280.1178\_7.35 | 1.50E+09 | 2.20E+09 | 1.50E+09 | 9.00E+08 | 8.00E+08 | 5.40E+08 | 7.10E+06 | ND |
| | 280.1179\_4.82 | 3.10E+09 | 1.30E+09 | 1.20E+09 | 1.80E+09 | 2.90E+08 | 1.50E+08 | 1.60E+07 | ND |
| | 280.1179\_5.38 | 8.80E+08 | 6.30E+08 | 8.80E+08 | 8.00E+08 | 1.00E+09 | 7.90E+08 | 8.20E+07 | ND |
| | 280.1180\_4.55 | 5.00E+08 | 3.80E+08 | 2.40E+08 | 5.90E+08 | 1.70E+08 | 8.20E+07 | ND | ND |
| | 280.1180\_4.92 | 5.50E+08 | 2.10E+08 | 2.30E+08 | 3.60E+08 | 5.90E+07 | 4.00E+07 | ND | ND |
| | 280.1180\_6.55 | 1.30E+09 | 2.70E+09 | 1.10E+09 | 1.90E+09 | 7.00E+08 | 4.00E+08 | 2.20E+08 | ND |
| | 280.1180\_6.90 | 2.30E+08 | 2.20E+08 | 2.70E+08 | 1.20E+08 | 1.40E+08 | 1.70E+08 | 1.20E+07 | ND |
| | 280.1181\_6.22 | 2.40E+08 | 1.00E+08 | 2.90E+08 | 1.80E+08 | 1.60E+08 | 2.70E+08 | 2.20E+07 | ND |
| C14H19NO5 | 282.1331\_4.20 | 3.50E+07 | 2.50E+07 | 5.20E+07 | 4.80E+07 | 2.00E+07 | 1.70E+07 | 2.30E+06 | ND |
| | 282.1331\_7.33 | 1.40E+07 | 3.20E+06 | 2.20E+07 | 1.90E+07 | 4.60E+07 | 5.00E+07 | ND | ND |
| | 282.1331\_7.49 | 4.40E+07 | 2.10E+08 | 1.00E+08 | 8.20E+07 | 1.80E+08 | 1.10E+08 | 4.10E+06 | ND |
| | 282.1332\_5.49 | 1.80E+08 | 1.10E+08 | 2.90E+08 | 5.70E+08 | 4.80E+08 | 1.70E+08 | 4.10E+06 | ND |
| | 282.1333\_4.93 | 1.30E+09 | 1.20E+09 | 5.60E+08 | 2.20E+09 | 3.20E+09 | 1.20E+09 | ND | ND |
| | 282.1333\_8.58 | 1.40E+08 | 8.00E+07 | 8.50E+07 | 9.10E+07 | 7.80E+07 | 8.80E+07 | 2.40E+06 | ND |
| | 282.1334\_6.25 | 2.70E+07 | 4.00E+07 | 1.10E+08 | 1.30E+08 | 8.30E+07 | 5.40E+07 | 1.70E+08 | ND |
| | 282.1335\_4.76 | 3.90E+08 | 3.80E+08 | 2.20E+08 | 1.10E+09 | 1.10E+09 | 6.00E+08 | ND | ND |
| | 282.1336\_6.40 | 2.50E+09 | 2.20E+09 | 1.10E+09 | 1.30E+09 | 6.90E+08 | 3.80E+08 | 1.40E+07 | ND |
| C14H19NO6 | 298.1281\_5.07 | 1.10E+09 | 9.50E+08 | 8.80E+08 | 9.00E+08 | 4.20E+08 | 3.00E+08 | 6.70E+07 | ND |
| | 298.1283\_3.44 | 1.20E+08 | 9.90E+07 | 4.80E+07 | 1.20E+08 | 1.90E+07 | 8.60E+06 | ND | ND |
| | 298.1283\_3.75 | 8.40E+08 | 4.10E+08 | 3.50E+08 | 6.60E+08 | 1.50E+08 | 7.30E+07 | 6.40E+06 | ND |
| | 298.1283\_4.37 | 1.70E+08 | 1.10E+08 | 6.40E+07 | 9.50E+07 | 2.70E+07 | 2.20E+07 | ND | ND |
| | 298.1285\_4.04 | 2.70E+08 | 1.10E+08 | 1.30E+08 | 1.90E+08 | 6.70E+07 | 4.10E+07 | ND | ND |
| | 298.1285\_7.73 | 1.80E+08 | 2.10E+07 | 4.70E+07 | 6.20E+07 | 4.90E+07 | 2.80E+07 | ND | ND |
| | 298.1286\_4.69 | 9.10E+08 | 5.70E+08 | 1.30E+08 | 2.20E+08 | 4.20E+07 | 2.60E+07 | ND | ND |
| | 298.1287\_2.98 | 5.30E+07 | 1.50E+07 | 2.40E+07 | 3.90E+07 | 8.50E+06 | 2.90E+06 | ND | ND |
| | 298.1287\_5.88 | 3.10E+09 | 1.70E+09 | 1.00E+09 | 1.90E+09 | 3.40E+08 | 1.80E+08 | 3.00E+06 | ND |
| C14H21NO6 | 300.1439\_4.69 | 3.80E+08 | 2.70E+08 | 1.90E+08 | 1.00E+09 | 5.30E+08 | 1.90E+08 | ND | ND |
| | 300.1439\_7.29 | 1.80E+08 | 8.10E+07 | 1.40E+08 | 1.50E+08 | 9.80E+07 | 5.90E+07 | 2.90E+06 | ND |
| | 300.1440\_4.01 | 1.20E+08 | 5.10E+07 | 7.70E+07 | 4.90E+08 | 1.40E+08 | 3.10E+07 | ND | ND |
| | 300.1440\_5.38 | 7.70E+08 | 4.30E+08 | 4.80E+08 | 1.60E+09 | 6.10E+08 | 4.40E+08 | 1.00E+08 | ND |
| | 300.1441\_4.45 | 3.20E+08 | 1.80E+08 | 2.20E+08 | 7.10E+08 | 2.40E+08 | 1.80E+08 | 4.30E+07 | ND |
| | 300.1443\_5.77 | 2.90E+09 | 2.20E+09 | 1.40E+09 | 1.80E+09 | 1.20E+09 | 8.10E+08 | ND | ND |
| Total | | 6.60E+10 | 6.20E+10 | 4.43E+10 | 6.00E+10 | 2.87E+10 | 1.80E+10 | 4.86E+09 | 3.99E+07 |
| \* triticone A/B | | | | | | | | | |
| ‡ cpaA product | | | | | | | | | |
| # triticone C or D | | | | | | | | | |
| ND not detected | | | | | | | | | |

## Slide 13
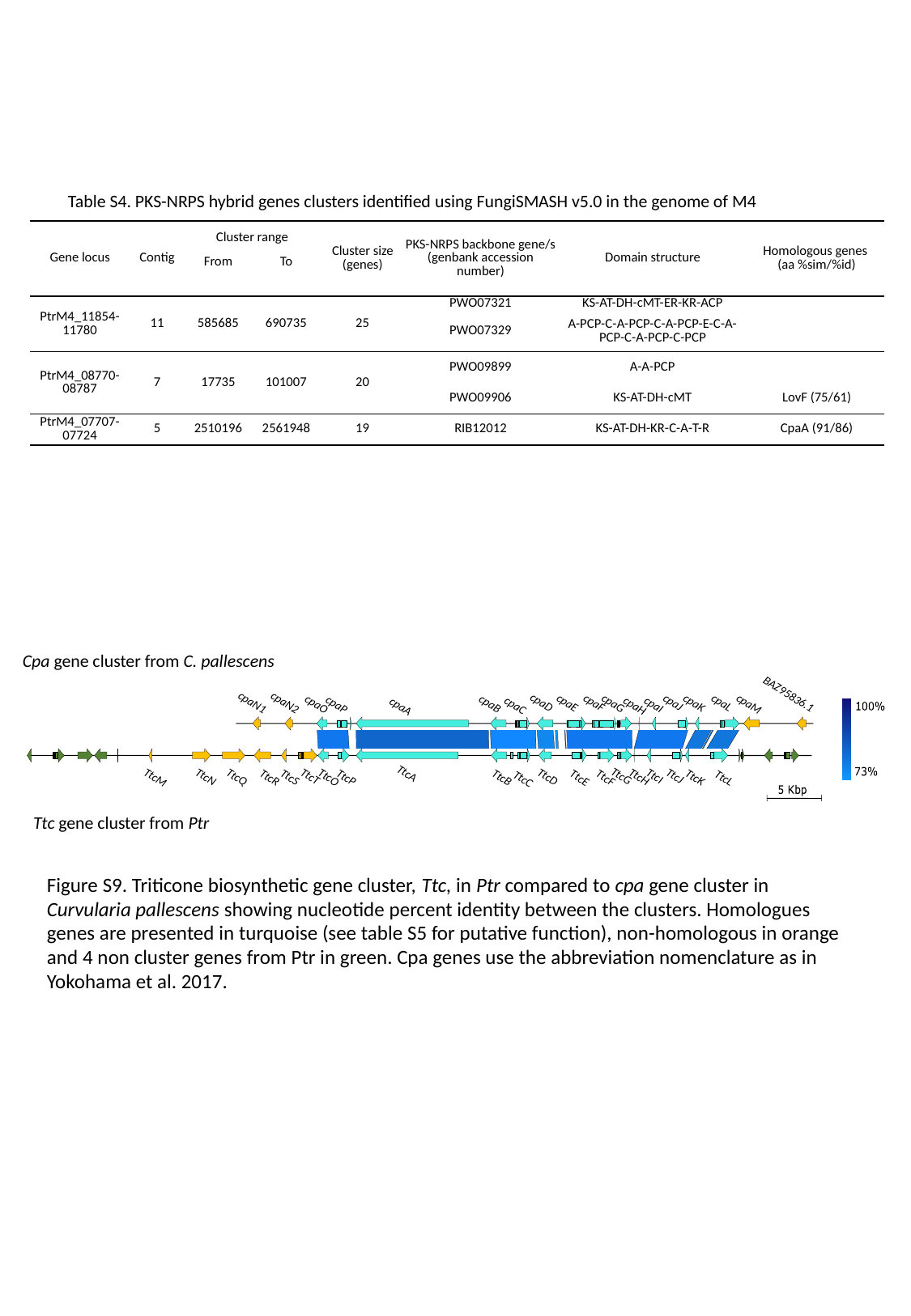

Table S4. PKS-NRPS hybrid genes clusters identified using FungiSMASH v5.0 in the genome of M4
| Gene locus | Contig | Cluster range | | Cluster size (genes) | PKS-NRPS backbone gene/s (genbank accession number) | Domain structure | Homologous genes (aa %sim/%id) |
| --- | --- | --- | --- | --- | --- | --- | --- |
| | | From | To | | | | |
| PtrM4\_11854-11780 | 11 | 585685 | 690735 | 25 | PWO07321 | KS-AT-DH-cMT-ER-KR-ACP | |
| | | | | | PWO07329 | A-PCP-C-A-PCP-C-A-PCP-E-C-A-PCP-C-A-PCP-C-PCP | |
| | | | | | | | |
| PtrM4\_08770-08787 | 7 | 17735 | 101007 | 20 | PWO09899 | A-A-PCP | |
| | | | | | PWO09906 | KS-AT-DH-cMT | LovF (75/61) |
| PtrM4\_07707-07724 | 5 | 2510196 | 2561948 | 19 | RIB12012 | KS-AT-DH-KR-C-A-T-R | CpaA (91/86) |
Cpa gene cluster from C. pallescens
BAZ95836.1
cpaJ
cpaN2
cpaL
cpaF
cpaD
cpaE
cpaN1
cpaK
cpaG
cpaB
cpaM
cpaO
cpaP
cpaI
cpaC
cpaH
cpaA
TtcA
TtcJ
TtcG
TtcI
TtcT
TtcN
TtcD
TtcH
TtcS
TtcQ
TtcO
TtcM
TtcR
TtcK
TtcB
TtcF
TtcE
TtcP
TtcL
TtcC
Ttc gene cluster from Ptr
Figure S9. Triticone biosynthetic gene cluster, Ttc, in Ptr compared to cpa gene cluster in Curvularia pallescens showing nucleotide percent identity between the clusters. Homologues genes are presented in turquoise (see table S5 for putative function), non-homologous in orange and 4 non cluster genes from Ptr in green. Cpa genes use the abbreviation nomenclature as in Yokohama et al. 2017.

## Slide 14
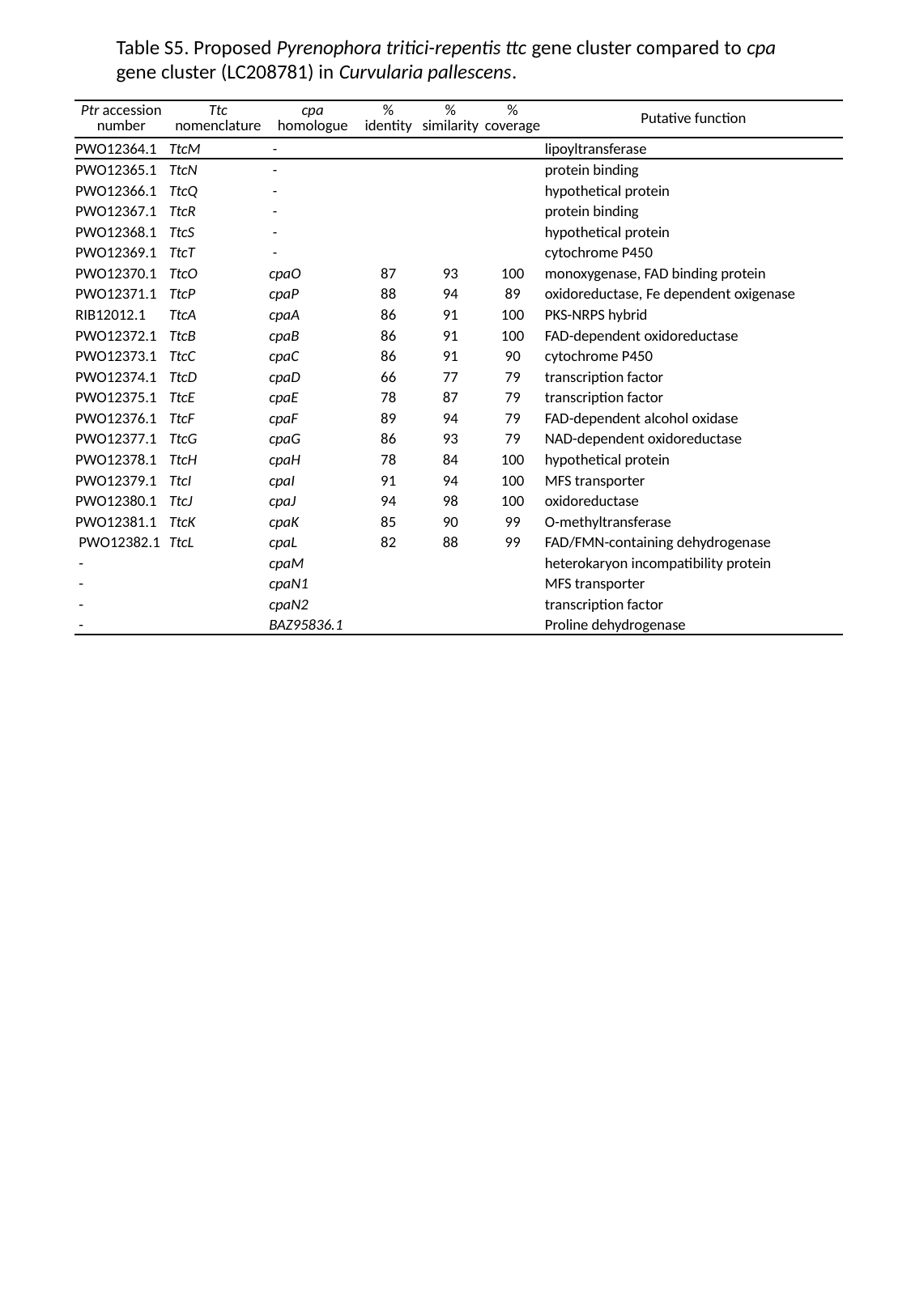

Table S5. Proposed Pyrenophora tritici-repentis ttc gene cluster compared to cpa gene cluster (LC208781) in Curvularia pallescens.
| Ptr accession number | Ttc nomenclature | cpa homologue | % identity | % similarity | % coverage | Putative function |
| --- | --- | --- | --- | --- | --- | --- |
| PWO12364.1 | TtcM | - | | | | lipoyltransferase |
| PWO12365.1 | TtcN | - | | | | protein binding |
| PWO12366.1 | TtcQ | - | | | | hypothetical protein |
| PWO12367.1 | TtcR | - | | | | protein binding |
| PWO12368.1 | TtcS | - | | | | hypothetical protein |
| PWO12369.1 | TtcT | - | | | | cytochrome P450 |
| PWO12370.1 | TtcO | cpaO | 87 | 93 | 100 | monoxygenase, FAD binding protein |
| PWO12371.1 | TtcP | cpaP | 88 | 94 | 89 | oxidoreductase, Fe dependent oxigenase |
| RIB12012.1 | TtcA | cpaA | 86 | 91 | 100 | PKS-NRPS hybrid |
| PWO12372.1 | TtcB | cpaB | 86 | 91 | 100 | FAD-dependent oxidoreductase |
| PWO12373.1 | TtcC | cpaC | 86 | 91 | 90 | cytochrome P450 |
| PWO12374.1 | TtcD | cpaD | 66 | 77 | 79 | transcription factor |
| PWO12375.1 | TtcE | cpaE | 78 | 87 | 79 | transcription factor |
| PWO12376.1 | TtcF | cpaF | 89 | 94 | 79 | FAD-dependent alcohol oxidase |
| PWO12377.1 | TtcG | cpaG | 86 | 93 | 79 | NAD-dependent oxidoreductase |
| PWO12378.1 | TtcH | cpaH | 78 | 84 | 100 | hypothetical protein |
| PWO12379.1 | TtcI | cpaI | 91 | 94 | 100 | MFS transporter |
| PWO12380.1 | TtcJ | cpaJ | 94 | 98 | 100 | oxidoreductase |
| PWO12381.1 | TtcK | cpaK | 85 | 90 | 99 | O-methyltransferase |
| PWO12382.1 | TtcL | cpaL | 82 | 88 | 99 | FAD/FMN-containing dehydrogenase |
| - | | cpaM | | | | heterokaryon incompatibility protein |
| - | | cpaN1 | | | | MFS transporter |
| - | | cpaN2 | | | | transcription factor |
| - | | BAZ95836.1 | | | | Proline dehydrogenase |

## Slide 15
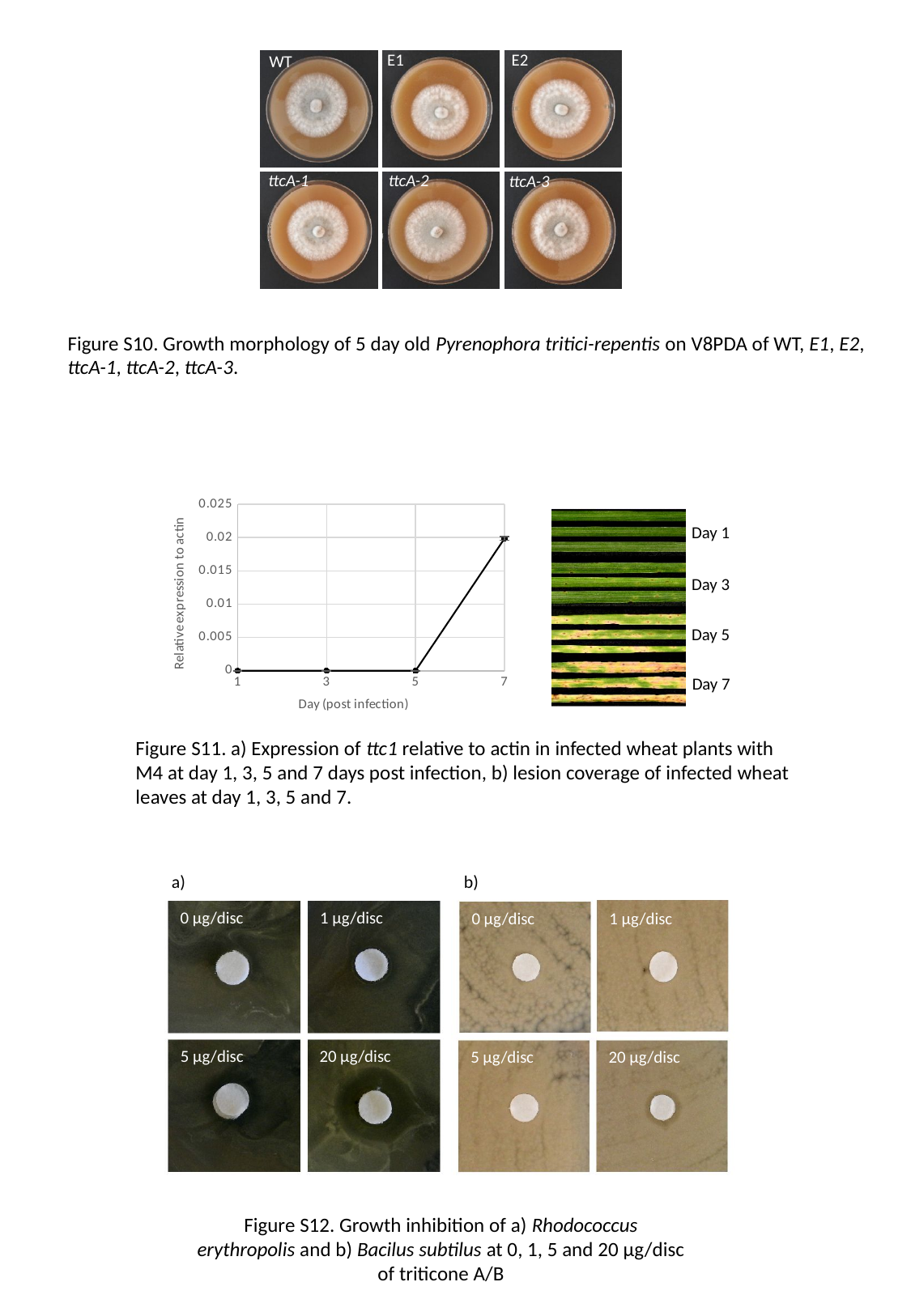

E1
E2
WT
ttcA-2
ttcA-1
ttcA-3
Figure S10. Growth morphology of 5 day old Pyrenophora tritici-repentis on V8PDA of WT, E1, E2, ttcA-1, ttcA-2, ttcA-3.
### Chart
| Category | |
|---|---|
Day 1
Day 3
Day 5
Day 7
Figure S11. a) Expression of ttc1 relative to actin in infected wheat plants with M4 at day 1, 3, 5 and 7 days post infection, b) lesion coverage of infected wheat leaves at day 1, 3, 5 and 7.
a)
b)
0 µg/disc
1 µg/disc
0 µg/disc
1 µg/disc
20 µg/disc
5 µg/disc
5 µg/disc
20 µg/disc
Figure S12. Growth inhibition of a) Rhodococcus erythropolis and b) Bacilus subtilus at 0, 1, 5 and 20 µg/disc of triticone A/B

## Slide 16
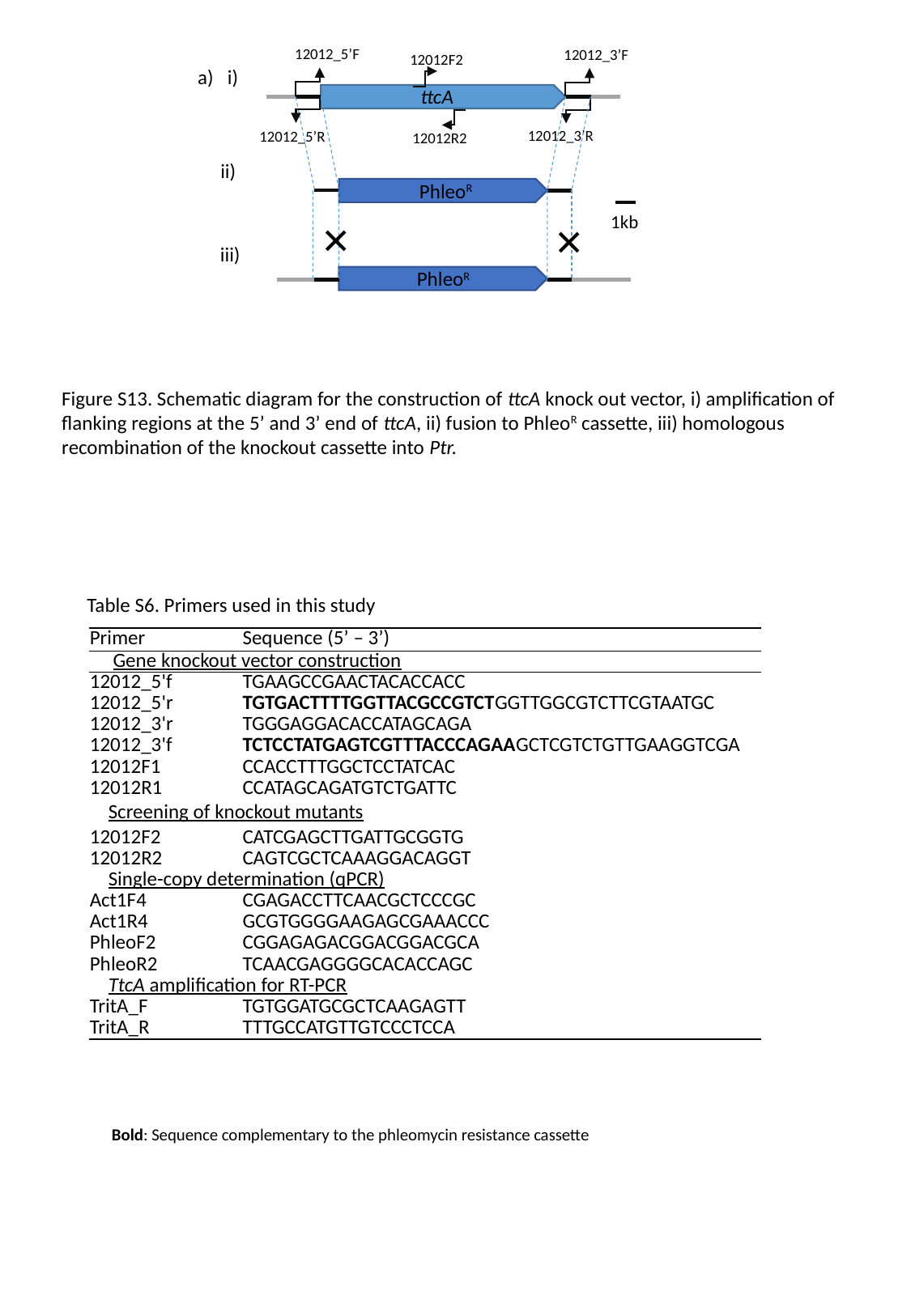

12012_5’F
12012_3’F
12012F2
a) i)
ttcA
12012_3’R
12012_5’R
12012R2
ii)
PhleoR
1kb
×
×
iii)
PhleoR
Figure S13. Schematic diagram for the construction of ttcA knock out vector, i) amplification of flanking regions at the 5’ and 3’ end of ttcA, ii) fusion to PhleoR cassette, iii) homologous recombination of the knockout cassette into Ptr.
Table S6. Primers used in this study
| Primer | Sequence (5’ – 3’) |
| --- | --- |
| Gene knockout vector construction | |
| 12012\_5'f | TGAAGCCGAACTACACCACC |
| 12012\_5'r | TGTGACTTTTGGTTACGCCGTCTGGTTGGCGTCTTCGTAATGC |
| 12012\_3'r | TGGGAGGACACCATAGCAGA |
| 12012\_3'f | TCTCCTATGAGTCGTTTACCCAGAAGCTCGTCTGTTGAAGGTCGA |
| 12012F1 | CCACCTTTGGCTCCTATCAC |
| 12012R1 | CCATAGCAGATGTCTGATTC |
| Screening of knockout mutants | |
| 12012F2 | CATCGAGCTTGATTGCGGTG |
| 12012R2 | CAGTCGCTCAAAGGACAGGT |
| Single-copy determination (qPCR) | |
| Act1F4 | CGAGACCTTCAACGCTCCCGC |
| Act1R4 | GCGTGGGGAAGAGCGAAACCC |
| PhleoF2 | CGGAGAGACGGACGGACGCA |
| PhleoR2 | TCAACGAGGGGCACACCAGC |
| TtcA amplification for RT-PCR | |
| TritA\_F | TGTGGATGCGCTCAAGAGTT |
| TritA\_R | TTTGCCATGTTGTCCCTCCA |
Bold: Sequence complementary to the phleomycin resistance cassette
